# Relations between neurometabolism and clinical biomarkers in patients with metabolic disease

**DOI:** 10.3389/fnins.2025.1547010

**Published:** 2025-04-15

**Authors:** Chao-Chao Chen, Ming-Shi Tan, Jiang-Tao Yin, Jian-Ming Li, Ying Li

**Affiliations:** ^1^Department of Pathology, Sun Yat-Sen Memorial Hospital, Sun Yat-Sen University, Guangzhou, China; ^2^Department of Pathology, Soochow University Medical School, Suzhou, China

**Keywords:** PET-CT, hypertension, T2DM, obesity, gout, clinical biomarkers

## Abstract

The global prevalence of metabolic diseases, including hypertension, type 2 diabetes mellitus (T2DM), gout, and obesity, has significantly increased over the past two decades. The brain plays a central role in regulating both human behavior and metabolism. Understanding the potential connections among these metabolic diseases and the involvement of the brain in their progression presents an intriguing and critical area of research. In this study, we analyzed PET-CT images and clinical biomarkers from 112 cases of hypertension, 56 cases of T2DM, 11 cases of obesity, and 14 cases of gout. Standardized uptake value ratios (SUVRs) were extracted from various brain regions using the Spatial-Normalization-of-Brain-PET-Images (SNBPI) software. The SUVRs were calculated using the standard methodology, where the mean standardized uptake value (SUV) of each region of interest (ROI) was divided by the mean SUV of the reference region, that is the whole cerebellum. The SNBPI tool was employed for intensity normalization. Partial correlation analysis was conducted to examine the relationships between SUVRs in different brain regions and clinical biomarkers, adjusting for sex, age, and BMI. Brain network metabolic connectivity was assessed using Permutation_IHEP software and visualized with BrainNet Viewer. Our results indicate that SUVRs in most brain regions were decreased in patients with hypertension or T2DM but increased in patients with obesity or gout. Specifically, SUVRs in brain regions associated with blood pressure were correlated with blood uric acid, creatinine, potassium, and apolipoprotein B. SUVRs in brain regions related to blood glucose were associated with blood triglycerides and cholinesterase. SUVRs in BMI-related brain regions correlated with blood urea nitrogen, aspartate aminotransferase, and alkaline phosphatase. SUVRs in brain regions associated with gout were correlated with fasting blood glucose, glutamic oxalacetic transaminase, total bilirubin, and alkaline phosphatase. Furthermore, brain network metabolic connectivity was reduced in patients with hypertension, T2DM, or obesity but increased in patients with gout. Our findings suggest that uric acid may negatively relate with blood pressure and glucose levels, while blood glucose and blood lipid levels may be positively correlated with each other. Gout appears distinct from other metabolic diseases and may offer a protective effect on brain function. The right superior parietal gyrus may be implicated in impaired renal function during the progression of hypertension. The left precentral gyrus and bilateral middle frontal gyri may relate to dyslipidemia and the potential development of atherosclerotic cardiovascular disease in patients with T2DM. In conclusion, our study highlights potential relationships among metabolic diseases and suggests the possible regulatory roles of specific brain regions in the progression of these conditions. These insights could pave the way for novel therapeutic strategies targeting brain metabolism in the management of metabolic diseases.

## Graphical abstract

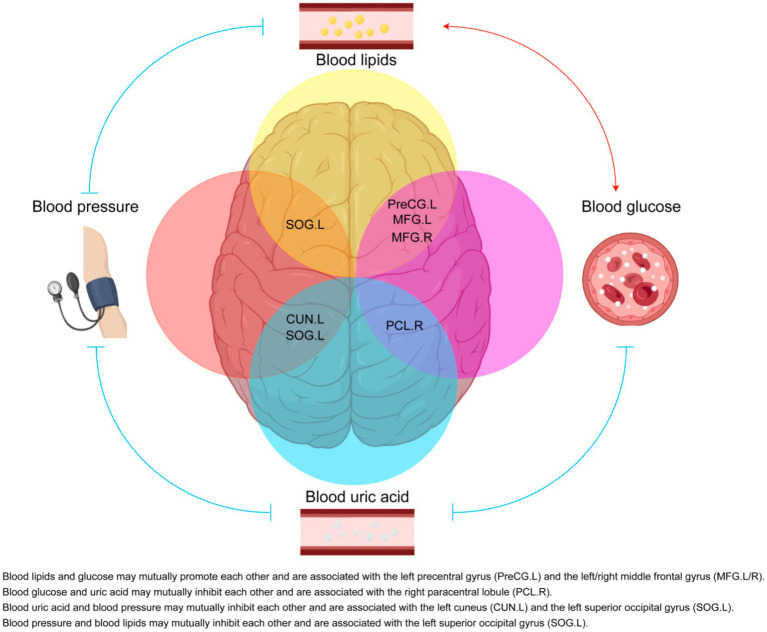

## 1 Introduction

Metabolic diseases, including hypertension, type 2 diabetes mellitus (T2DM), non-alcoholic fatty liver disease (NAFLD), gout, and obesity, have seen a marked increase in global prevalence over the past two decades, posing a significant burden on public health (Chew et al., 2023; Dalbeth et al., 2021). The brain plays a central role in regulating key physiological processes such as eating behavior (Watts et al., 2022), blood pressure (Kelly and Rothwell, 2020), and glucose homeostasis (Alonge et al., 2021). Notably, metabolic disorders have been linked to neurocognitive impairments, with hypertension (Lande and Kupferman, 2019), high body mass index (BMI) (Tanaka et al., 2020), and T2DM (Biessels and Despa, 2018) contributing to cognitive decline and increased risk of mental disorders. Conversely, gout and hyperuricemia have been suggested to exert a protective effect against neurodegenerative conditions such as Alzheimer’s disease (Wang et al., 2023). Additionally, obesity is associated with an increased risk of other metabolic disorders (Seravalle and Grassi, 2017; Kahn et al., 2006), and comorbidities such as hypertension and T2DM frequently co-occur (Ferrannini and Cushman, 2012). Despite these interconnections, the precise neural mechanisms underlying the relationships among metabolic diseases remain poorly understood.

Neuroimaging techniques, including functional magnetic resonance imaging (fMRI) and positron emission tomography-computed tomography (PET-CT), provide valuable insights into brain function in metabolic disorders. Prior fMRI studies have identified alterations in functional connectivity associated with cognitive dysfunction and microstructural integrity in hypertensive patients (Carnevale et al., 2020), as well as reduced activation in task-related brain regions in individuals with T2DM (Macpherson et al., 2017). Similarly, obesity has been linked to disrupted fronto-mesolimbic circuitry (Li et al., 2023), while PET studies have revealed widespread cortical hypometabolism in patients with treated hypertension. PET studies also indicate a strong trend toward hypometabolism throughout the cortex in patients with treated hypertension (Salerno et al., 1995). However, the extent to which the brain regulates metabolic disease progression and the potential shared neural substrates among these conditions remain unclear.

Our study aims to investigate the neural correlates of metabolic diseases by analyzing PET-CT imaging and clinical biomarkers from patients. Specifically, we aim to examine hypertension, T2DM, obesity, gout cases. By assessing neurometabolic patterns and their associations with clinical parameters, we seek to elucidate potential neural mechanisms underlying metabolic disease regulation. Our findings may provide novel insights into the brain’s role in modulating renal function in hypertension, dyslipidemia in T2DM, and the potential protective effects of uric acid on liver function and glucose homeostasis.

## 2 Materials and methods

### 2.1 Study design

We collected PET-CT images and clinical biomarkers, including blood pressure and blood biochemical indicators of hypertension, T2DM, obesity or gout patients. We extracted standardized uptake value ratios (SUVRs) of different brain regions and analyzed the differences of the SUVRs between normal patients and patients with above metabolic disease. Then we analyzed the correlations of the SUVRs with clinical biomarkers in patients. At last, we analyzed brain network metabolic connectivity changes in patients with above metabolic disease.

### 2.2 Participants recruitment

We collected the PET-CT images of 690 patients who underwent PET/CT in Sun Yat-sen Memorial Hospital during January 2018 and December 2023, after excluding those with malignant tumors, brain atrophy, cerebral ischemia and trauma, epilepsy, brain tumors, brain surgery, and psychiatric disorders. Patients with missing medical history or brain imaging data were also excluded. The ages of them were between 18 and 90 years, including both males and females. Based on outpatient or inpatient medical records, we identified 112 cases of hypertension (systolic/diastolic blood pressure ≥ 140/90 mmHg at least three times at different days), 56 cases of T2DM (typical symptoms of diabetes with random blood glucose ≥11.1 mmol/L, or fasting blood-glucose ≥7.0 mmol/L, or blood glucose after a glucose load ≥11.1 mmol/L, or HbA1c ≥ 6.5%), 11 cases of obesity (BMI ≥ 30 kg/m^2^), 14 cases of gout (blood uric acid ≥420 μmol/L and deposition of sodium uric acid crystals in joints). There were 497 patients without the aforementioned diseases (mostly due to pneumonia, hepatitis, thyroid disease, fractures, etc.) (Supplementary Table 4). In addition to PET/CT images, we collected the blood pressure and the data of 28 blood biochemical indicators within 1 week of the PET/CT examination, including blood sodium, blood potassium, blood chloride, blood calcium, urea, creatinine, uric acid, fasting blood glucose, total protein, albumin, globulin, alanine aminotransferase, aspartate aminotransferase, total bilirubin, direct bilirubin, indirect bilirubin, alkaline phosphatase, cholinesterase, urea nitrogen/creatinine, triglycerides, total cholesterol, high-density lipoprotein cholesterol, low-density lipoprotein cholesterol, apolipoprotein A1, apolipoprotein B, apolipoprotein E, free fatty acids, and total bile acids. The data collection was approved by the Medical Ethics Committee of Sun Yat-Sen memorial hospital, Sun Yat-Sen University (Approval ID: SYSKY-2024-706-01).

### 2.3 PET-CT image acquisition

Patients were fasted for at least 6 h before the PET-CT scanning. An intravenous catheter was placed into the forearm vein, and the arm was placed on a 44°C warmer. ^18^F-fluorodeoxyglucose (FDG) was injected into the patient’s vein. Participants rested for approximately 60 min in a quiet, dimly lit room before undergoing PET-CT scanning to minimize physiological variability. After resting, patients underwent PET/CT (Siemens Biography mCT) examination. In a dark and quiet environment, each participant’s head was placed on a headrest and gently secured with a strap. The scanning would last 20–30 min. CT brain images (CT parameters: 120 kV, 84 Eff.mAs, 300 mm FoV, 3.0 mm slice thickness, 1.5 mm increment, H31s medium smooth reconstruction kernel) and PET brain images (PET parameters: TrueX+TOF reconstruction method, 3 iterations/21 subsets, 5 mm FWHM Gaussian filter) were acquired in the same bed position for 1.5 min. PET brain images were then performed with attenuation and scatter correction. The MEMRS nuclear medicine image processing and reporting workstation (MedEx) was then used to process and analyze PET and CT images.

### 2.4 Preprocessing and analysis of PET/CT scanning images

PET brain images were converted into 3D nii format using the dcm2niigui.exe plugin in Mricron software, and then processed in MATLAB. We used SPM12 implemented in MATLAB software 2022b (MathWorks, Inc.) to process and analyze ^18^F-FDG-PET images. First, we used the ‘Pre-processing’ tool in the petpve12 toolkit of SPM12 to process the image centroid, by clicking ‘Set image origin to the Center-Of-Mass’ to set the centroid of all patients’ PET brain images to the midpoint of the anterior commissure (AC) line. Secondly, we used the adaptive probability brain atlas-based PET brain image unified spatial normalization method to spatially standardize the PET brain images processed in the first step, by normalizing all patients’ PET brain images to the Montreal Neurological Institute (MNI) space using the ‘Old Normalise: Estimate & Write’ method in SPM12 (this step requires batch processing with scripts in MATLAB). Thirdly, in SPM12, we clicked ‘Smooth’ and used an 8-mm full-width at half-maximum (FWHM) isotropic Gaussian kernel to smooth all PET brain images processed in the second step. Fourthly, after spatial normalization and smoothing of all patients’ PET brain images with SPM12, intensity normalization was performed on all patients’ PET brain images using the Spatial-Normalization-of-Brain-PET-Images (SNBPI)^1^ (Zhang et al., 2022), by selecting the entire cerebellum as the reference brain region to generate SUVR images. Fifthly, using the AAL brain atlas containing 90 brain regions (Tzourio-Mazoyer et al., 2002), SNBPI was used to extract SUVRs from different brain regions of normal, T2DM, hypertension, gout, and obesity patients. SUVRs is a normalized metric used to quantify radiotracer uptake in PET imaging, calculated by dividing the mean standardized uptake value (SUV) of a target region by the mean SUV of a reference region (SUVR = SUV_target_/SUV_reference_). This normalization approach is crucial for reducing inter-subject variability and enabling meaningful comparisons across individuals. The whole cerebellum (including both gray and white matter) was selected as the reference region, based on its widespread use and validation in previous literature (Liu et al., 2023; van Arendonk et al., 2023).

### 2.5 Brain network connectivity

#### 2.5.1 Establishment of brain network connectivity

Firstly, in SPM12, we clicked ‘Batch’, ‘SPM’, ‘Util’, and ‘3D to 4D File Conversion’ buttons step by step to convert the PET brain images of each group of patients to 4D nii format. Secondly, according to graph theory, we constructed the glucose metabolism network of each group as a set of nodes, with nodes representing brain regions and edges representing undirected connections between them. Nodes were defined as 90 brain anatomical regions using the AAL atlas with WFU-PickAtlas^2^ (Sun et al., 2020). The connectivity strength of the edges was calculated as the Pearson correlation coefficient of the average FDG SUVRs between each pair of nodes. Thirdly, the above weighted network was thresholded using sparsity (S) to construct a binary undirected network, with sparsity (S) being the ratio of existing edges to all possible edges. The sparsity of the sparsest network in the fully connected brain network was used.

#### 2.5.2 Modularity of brain network

Modularity analysis of brain network connectivity was performed using gretna software (Wang et al., 2015), by clicking ‘Network Analysis’ button to perform modularity analysis on nodes.

#### 2.5.3 Metabolic connectivity of brain network

Permutation tests were performed on the brain networks of disease and normal groups established under the AAL atlas using Permutation_IHEP software, with a setup of 5,000 permutations and a sparsity threshold of 0.35, the gout group had a sparsity threshold of 0.55 (the sparsest sparsity in the fully connected brain network) (Hsu et al., 2016; Bullmore and Sporns, 2009). Brain network metabolic connectivity was visualized using BrainNet Viewer (RRID:SCR_009446) (Xia et al., 2013).

### 2.6 Statistical analysis

In patients with hypertension, T2DM, and gout, the age-sex compositions were different from the control group. And we performed linear regression analysis on the extracted SUVRs using IBM SPSS Statistics 27, by setting gender and age as independent variables and SUVRs as dependent variable, to obtain the residual SUVR value. Then, we added the average SUVR value of the brain region in each group with the residual SUVR value to obtain the corrected SUVR value. Two-tailed independent Student’s t-tests or two-tailed independent Student’s t-tests with Welch’s correction were performed to analyze the differences between SUVRs of brain regions of normal and disease group (T2DM, hypertension, obesity, gout) using GraphPad Prism 9.5.0 to screen for differential brain regions (*p* < 0.05). Partial correlation analysis was performed on brain region SUVRs and blood biochemical indicators using IBM SPSS Statistics 27, with age, gender, and BMI as covariates, to screen for correlated indicators (*p* < 0.05). All results were expressed as mean ± SD, with n representing the number of independent biological replicates, and *p* value <0.05 considered significant. The test statistics and *p* values of partial correlation analysis are shown in Supplementary Table 2. The blood biochemical indicators of some patients were undetected within 1 week before the PET/CT examination and then were missing in our analysis.

## 3 Results

### 3.1 Recruitment and patient characteristics

We collected the PET-CT images of 690 patients without malignant tumors who underwent PET/CT in Sun Yat-sen Memorial Hospital during January 2018 and December 2023. According to their diagnosis, there were 112 cases of hypertension, 56 cases of T2DM, 11 cases of obesity, 14 cases of gout, and 497 cases without the above diseases. Baseline characteristics are presented in Supplementary Table 1. The clinical biomarkers were collected within 1 week before the PET/CT scanning. None of them have reported anxiety, depression, cognitive disorder, insomnia, or epilepsy.

A total of 497 cases without the metabolic diseases were set as control patients. Among these patients, most of them visited the hospital due to pneumonia, hepatitis, thyroid disease, fractures and so on (Supplementary Table 4). The overall cohort had a mean age of 53.37 ± 15.22 years, with a gender distribution of 52.52% males and 47.48% females. Among other cohorts, hypertensive patients (*n* = 112) showed a mean age of 63.02 ± 11.38 years (63.39% male), with blood pressure measurements of 167.55 ± 17.59 mmHg (systolic) and 97.62 ± 12.65 mmHg (diastolic), all of whom were treatment-naïve for at least 1 month. The T2DM group (*n* = 56) had a mean age of 59.68 ± 10.57 years (57.14% male) and fasting blood glucose levels of 6.56 ± 2.18 mmol/L, with all patients receiving regular metformin treatment. Obese patients (*n* = 11) demonstrated a mean age of 48.18 ± 12.38 years (45.45% male) and BMI of 33.19 ± 3.05, while the gout subgroup (*n* = 14) had a mean age of 58.57 ± 14.93 years (85.71% male), with both groups being medication-free.

### 3.2 Brain neurometabolism association to clinical cues in hypertension

**Figure 1:**
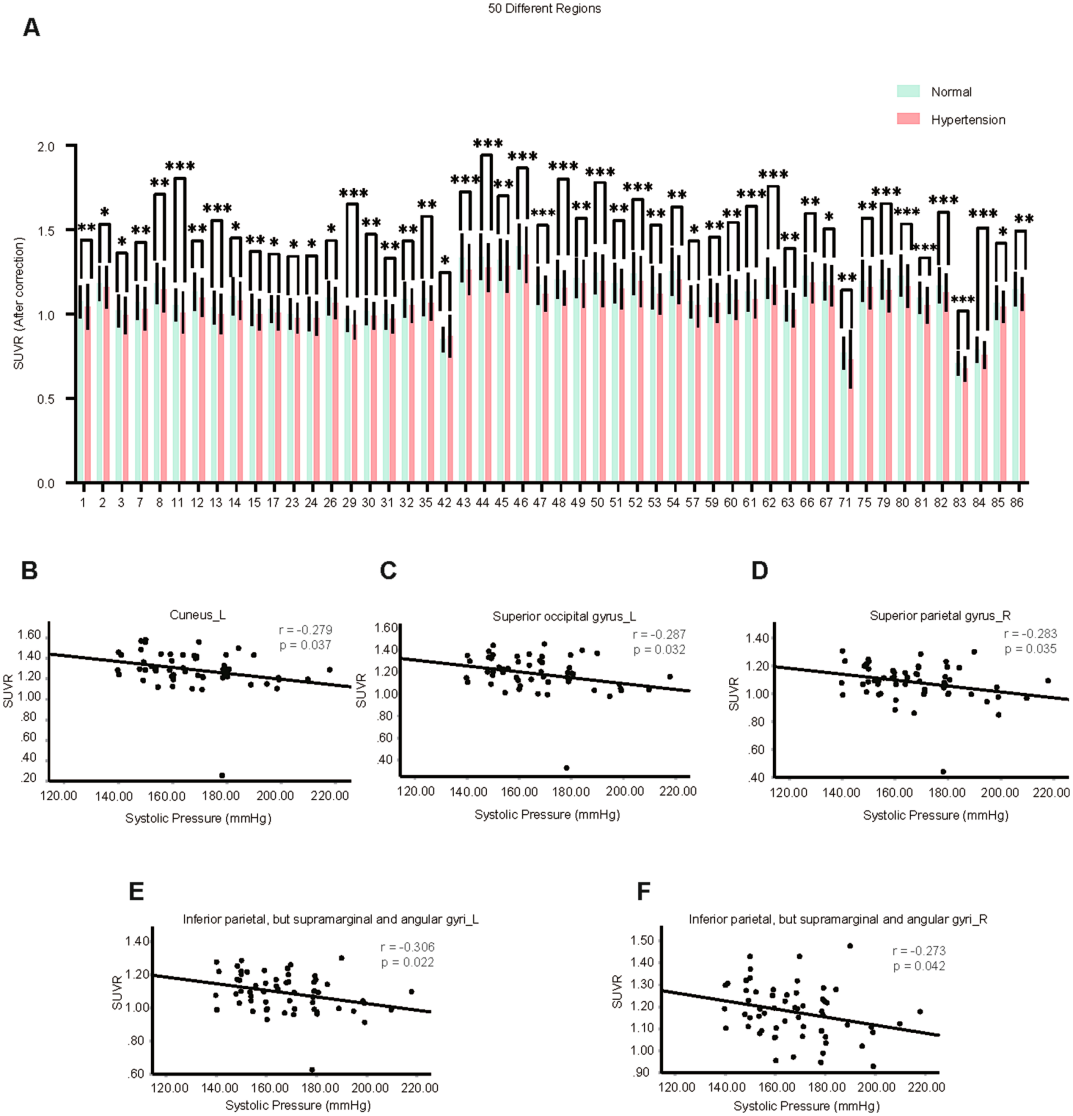
Brain neurometabolism association to clinical cues in hypertension. **(A)** The corrected SUVRs of different brain regions of control and hypertension patients were compared using two-tailed unpaired Student’s t test or two-tailed independent Student’s t-test with Welch’s correction. The different number indicate different brain region, which was interpreted in Supplementary Table 3. *N* = 497 cases for control patients. *N* = 112 cases for hypertension group. **(B–F)** The correlations between the SUVRs of different brain regions and systolic blood pressure in hypertension patients were analyzed using partial correlation analysis by setting age, sex, and BMI as control variables. *N* = 59 cases for hypertension group. Data shown were mean ± SD. *p < 0.05; **p < 0.01; ***p < 0.001.

Compared with control patients, the average age of hypertension patients was higher (*p* = 0.0001) and there were more males with hypertension (*p* = 0.033). Then we analyzed the SUVRs and the corrected SUVRs, which were adjusted according to the age and sex, of patients with hypertension. We found the SUVRs and the corrected SUVRs of 49 brain regions in the hypertension group were significantly decreased, mainly including the frontal lobe, occipital lobe, temporal lobe, bilateral insula, bilateral cuneus, left pallidum, and left caudate nucleus (Figure 1A and Supplementary Figure 1A). Interestingly, the SUVRs and the corrected SUVRs of right amygdala were increased in hypertension group (Figure 1A and Supplementary Figure 1A, number 42).

Among these 50 brain regions with changed SUVRs, the SUVRs of 5 brain regions in the hypertension group were negatively correlated with the measured highest systolic blood pressure using partial correlation analysis by setting age, gender, and BMI as covariates (Supplementary Table 2). These five brain regions were the left cuneus, left superior occipital gyrus, right superior parietal gyrus, bilateral supramarginal and angular gyri (Figures 1B–F). The results indicate these five brain regions might be associated with the blood pressure.

### 3.3 Brain neurometabolism association to metabolic biomarkers in hypertension

**Figure 2:**
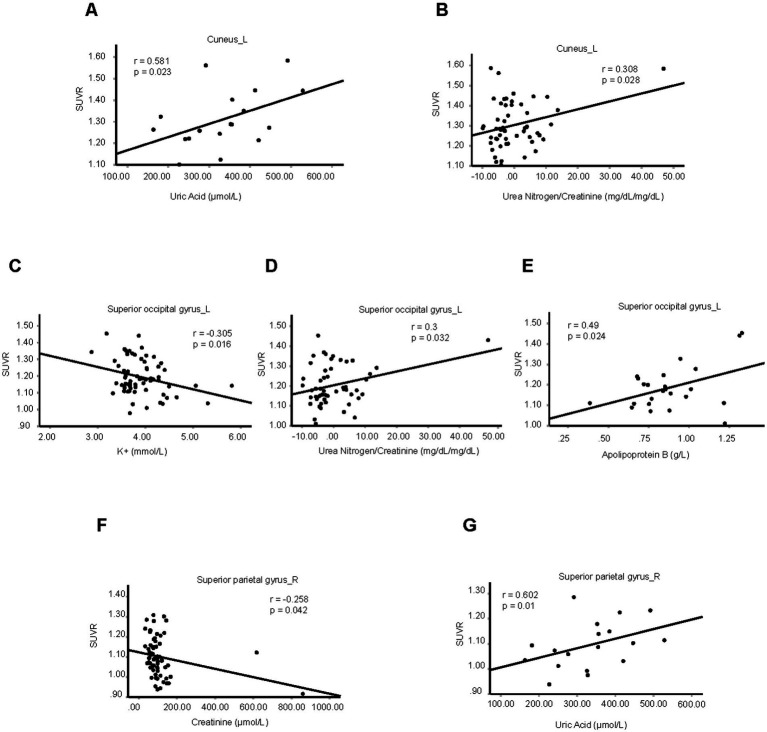
Brain neurometabolism association to metabolic biomarkers in hypertension. **(A–G)** The correlations between the SUVRs of different brain regions and blood biochemical indicators in hypertension patients were analyzed using partial correlation analysis by setting age, sex, and BMI as control variables. *N* = 18 cases in panel **(A)**, 54 cases in panel **(B)**, 65 cases in panel **(C)**, 54 cases in panel **(D)**, 24 cases in panel **(E)**, 66 cases in panel **(F)**, 18 cases in panel **(G)**.

We further analyzed the relation between SUVRs of these five brain regions with blood biochemical indicators in the hypertension group, including K^+^, Na^+^, Cl^−^, Ca^2+^, urea nitrogen, uric acid, creatinine, fasting blood-glucose, liver function tests, blood lipid examination, using partial correlation analysis by setting age, gender, and BMI as covariates (Supplementary Table 2). We found the SUVR of left cuneus was positively correlated with uric acid and urea nitrogen/creatinine. The SUVR of left superior occipital gyrus was negatively correlated with serum potassium, and positively correlated with urea nitrogen/creatinine and apolipoprotein B. The SUVR of right superior parietal gyrus was negatively correlated with creatinine and positively correlated with uric acid (Figures 2A–G).

Compared with control patients, the level of blood creatinine was increased significantly in hypertension group, but the value of uric acid, urea nitrogen/creatinine, serum potassium, and apolipoprotein B were unchanged in hypertension group (Supplementary Figures 2A–E). For control patients, the SUVR of left cuneus was positively correlated with uric acid but not urea nitrogen/creatinine (Supplementary Figures 2F–G). The SUVR of left superior occipital gyrus was not correlated with serum potassium, urea nitrogen/creatinine or apolipoprotein B (Supplementary Figures 2H–J). Also, the SUVR of right superior parietal gyrus showed no relations with creatinine or uric acid (Supplementary Figures 2K–L). These results indicate the impaired function of kidney in hypertension might be associated with right superior parietal gyrus.

### 3.4 Alterations in brain network connectivity in hypertension

**Figure 3:**
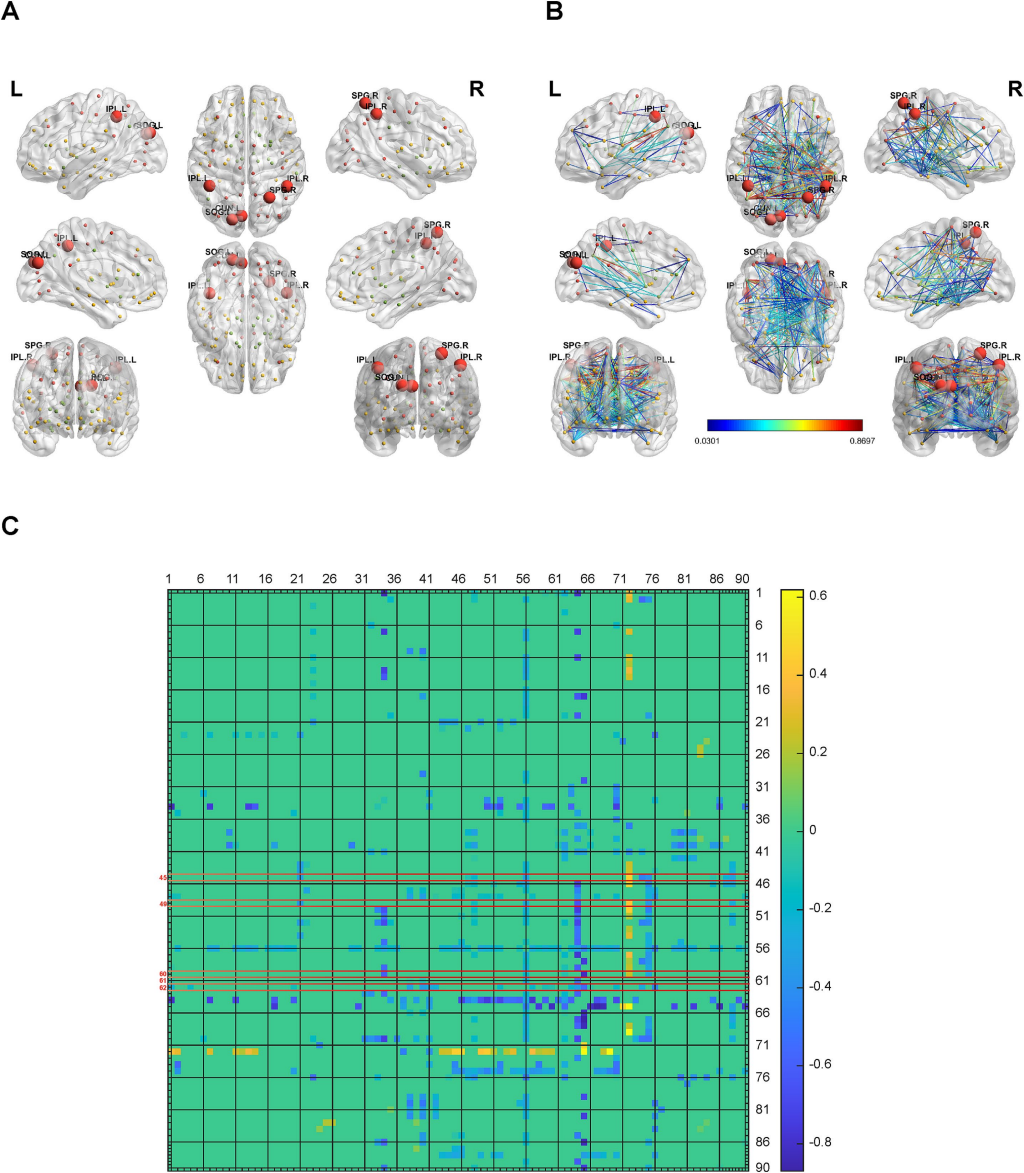
Alterations in brain network connectivity in hypertension. **(A)** Locations of the left cuneus, left superior occipital gyrus, right superior parietal gyrus, bilateral supramarginal and angular gyri in 3D brain were showed. **(B,C)** The changes of metabolic connectivity among different brain regions were showed. The changes in metabolic connectivity of the left cuneus, left superior occipital gyrus, right superior parietal gyrus, bilateral supramarginal and angular gyri with other brain regions were annotated in panel **(B)**, and labeled with red font in panel **(C)**. The different number indicate different brain region, which was interpreted in Supplementary Table 3. The color bar indicates the absolute value of magnitude changes in connectivity strength in panel **(B)** and magnitude changes in connectivity strength in panel **(C)**. *N* = 154 cases for control patients. *N* = 112 cases for hypertension group.

To analyze the brain network connectivity, we selected 154 cases of control patients, who had similar average ages (*p* = 0.4941) and sex compositions (*p* = 0.631) with patients with hypertension. Using BrainNet Viewer, we analyzed the brain network metabolic connectivity of patients with hypertension. We found most of the metabolic connections among the 90 brain regions were weakened in patients with hypertension, and the metabolic connections between the brain regions related to systolic blood pressure and other brain regions were also weakened (Figures 3A–C). The metabolic connectivity between most brain regions was weakened in the hypertension group compared with the control group (Figures 3A–C).

### 3.5 Brain neurometabolism association to clinical cues in T2DM

**Figure 4:**
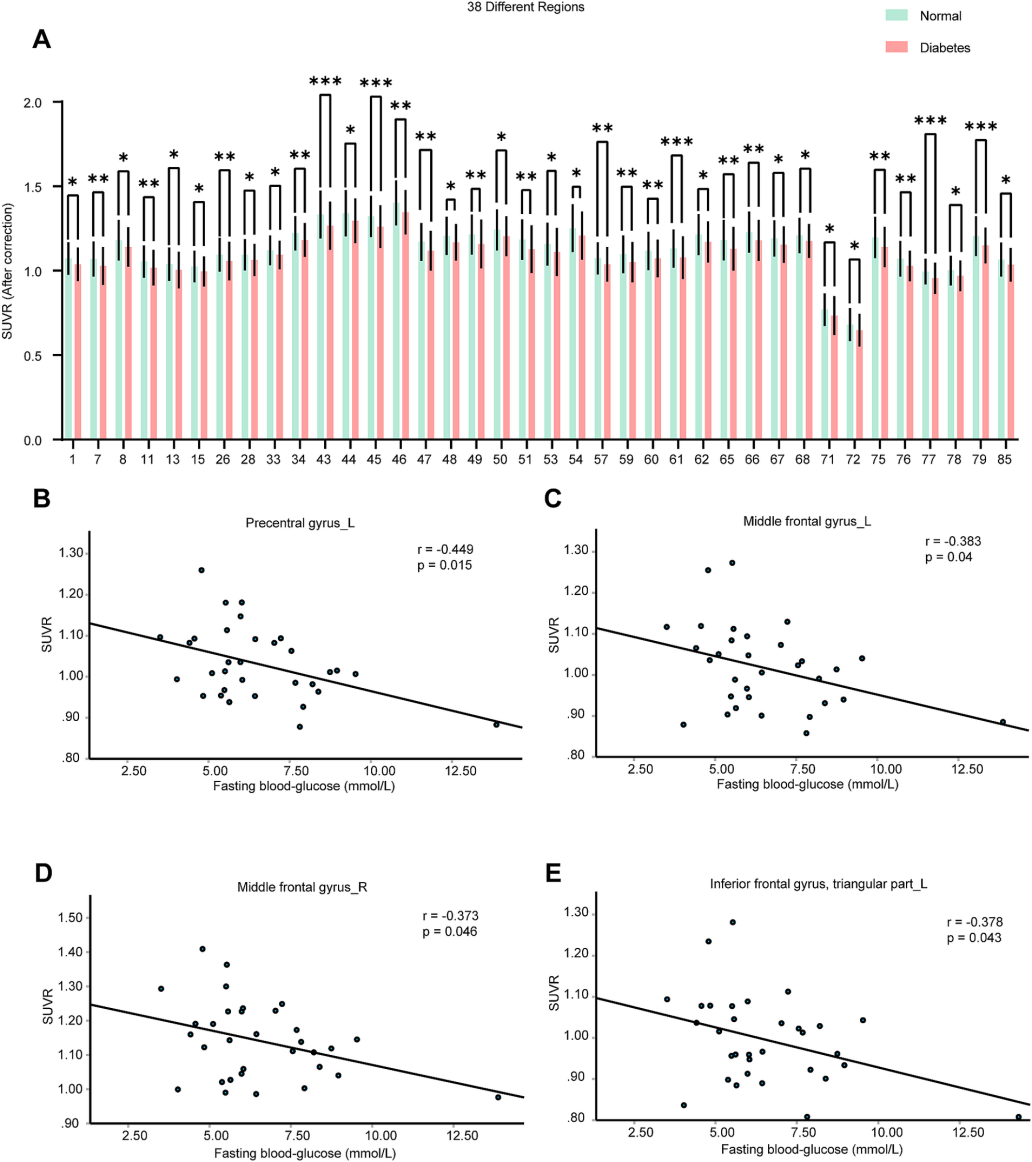
Brain neurometabolism association to clinical cues in T2DM. **(A)** The corrected SUVRs of different brain regions of control and T2DM patients were compared using two-tailed unpaired Student’s t test or two-tailed independent Student’s t-test with Welch’s correction. The different number indicate different brain region, which was interpreted in Supplementary Table 3. *N* = 497 cases for control patients. *N* = 56 cases for T2DM group. **(B–E)** The correlations between the SUVRs of different brain regions and fasting blood-glucose in T2DM patients were analyzed using partial correlation analysis by setting age, sex, and BMI as control variables. *N* = 32 cases for T2DM group. Data shown were mean ± SD. *p < 0.05; **p < 0.01; ***p < 0.001.

Compared with control patients, the average age of T2DM patients was higher (*p* = 0.0026) and the sex compositions were similar (*p* = 0.493). Then we analyzed the SUVRs and the corrected SUVRs, which were adjusted according to the age and sex, of patients with T2DM. Compared with control patients, the SUVRs and the corrected SUVRs of 38 brain regions in the T2DM group were significantly decreased, mainly including the frontal lobe, cingulate, occipital, parietal lobes, cuneus, precuneus, caudate nucleus, pallidum, and thalamus (Figure 4A and Supplementary Figure 3A). Among these 38 brain regions with changed SUVRs, the SUVRs of 4 brain regions in the T2DM group were negatively correlated with fasting blood-glucose using partial correlation analysis by setting age, gender, and BMI as covariates (Supplementary Table 2). These four brain regions were left precentral gyrus, the left middle frontal gyrus, the right middle frontal gyrus, and the left triangular part of inferior frontal gyrus (Figures 4B–E). The results indicate these four brain regions might be associated with the fasting blood-glucose in patients with T2DM.

For control patients, the SUVRs of left precentral gyrus, the bilateral middle frontal gyrus and the left triangular part of inferior frontal gyrus, were negatively correlated with fasting blood-glucose (Supplementary Figures 4A–D). The results indicate these brain regions might be associated with blood glucose physiologically.

### 3.6 Brain neurometabolism association to metabolic biomarkers in T2DM

**Figure 5:**
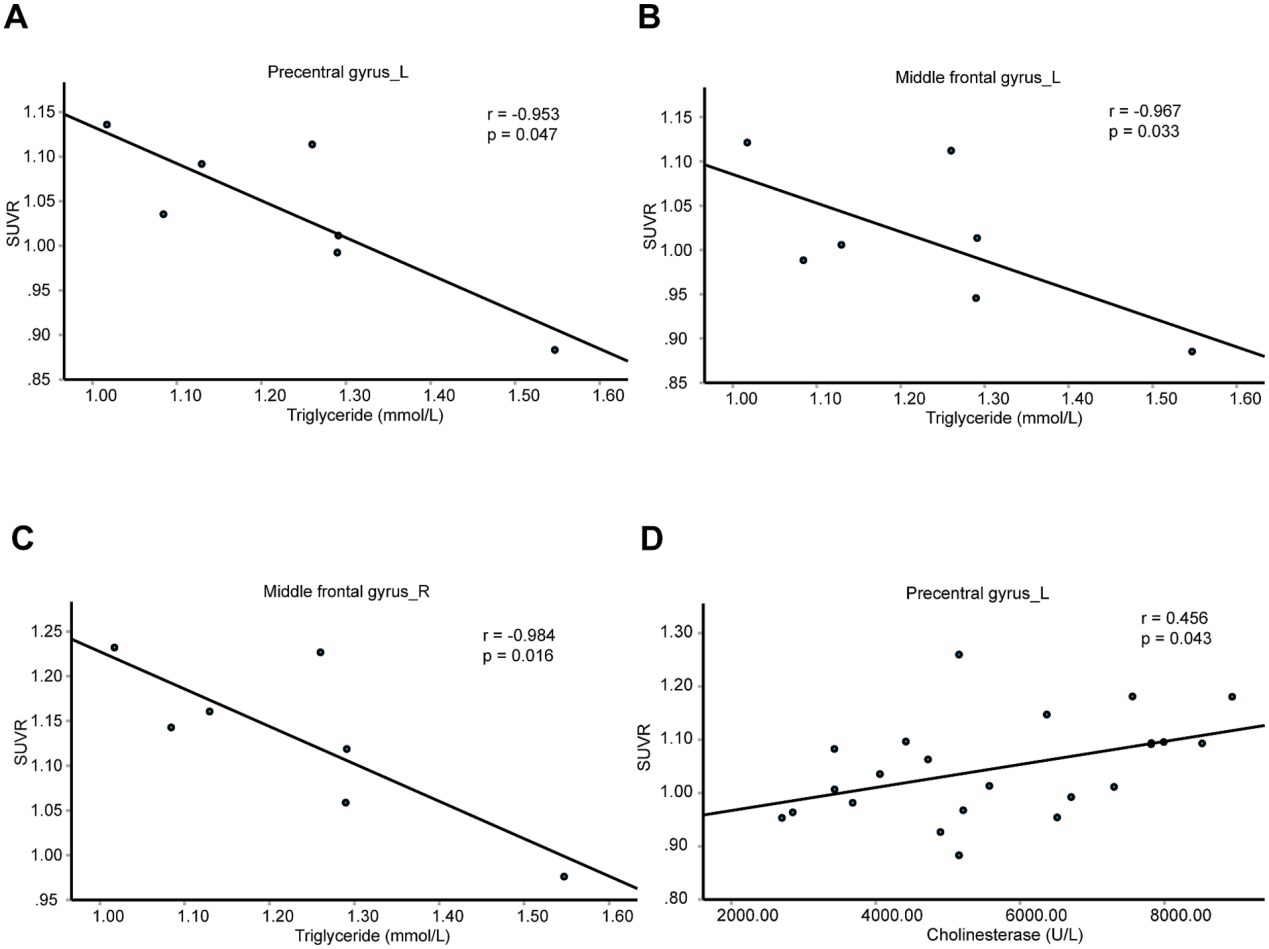
Brain neurometabolism association to metabolic biomarkers in T2DM. **(A–D)** The correlations between the SUVRs of different brain regions and blood biochemical indicators in T2DM patients were analyzed using partial correlation analysis by setting age, sex, and BMI as control variables. *N* = 7 cases in panels **(A–C)**. *N* = 23 cases in panel **(D)**.

We further analyzed the relations between SUVRs of these four brain regions with blood biochemical indicators in patients with T2DM, including K^+^, Na^+^, Cl^−^, Ca^2+^, urea nitrogen, uric acid, creatinine, liver function tests, blood lipid examination (Supplementary Table 2). We found the SUVRs of the left precentral gyrus and bilateral middle frontal gyrus were negatively correlated with triglycerides (Figures 5A–C). The SUVR of the left precentral gyrus was positively correlated with cholinesterase (Figure 5D). These results indicate the left precentral gyrus and bilateral middle frontal gyrus might be associated with blood glucose and lipid meantime. And left precentral gyrus might be associated with blood glucose and synthetic function of liver cells.

For control patients, the SUVRs of left precentral gyrus, the left middle frontal gyrus, the right middle frontal gyrus, and the left triangular part of inferior frontal gyrus were not correlated with triglycerides or cholinesterase (Supplementary Table 2).

### 3.7 Alterations in brain network connectivity in T2DM

**Figure 6:**
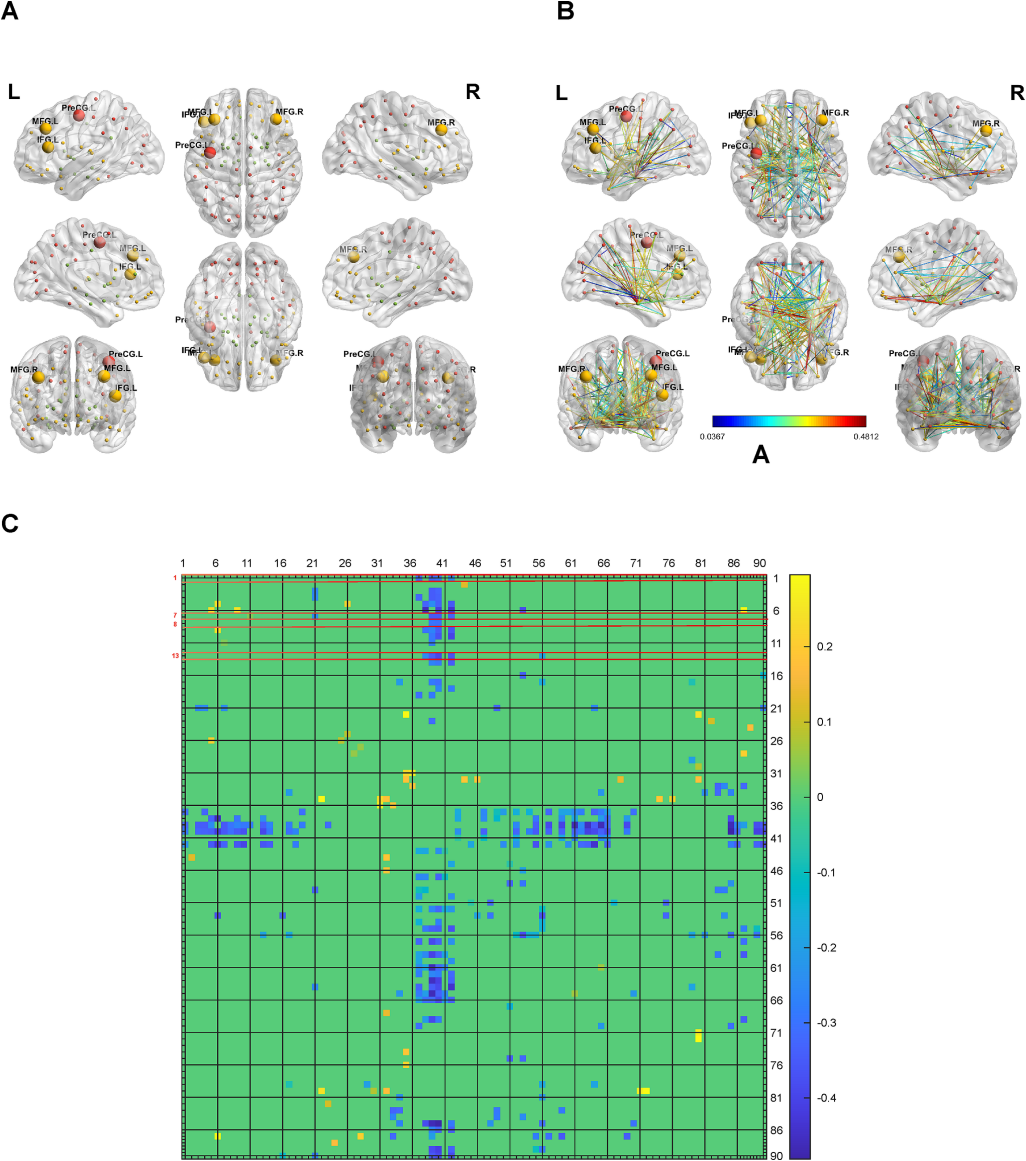
Alterations in brain network connectivity in T2DM. **(A)** Locations of the left precentral gyrus, the left middle frontal gyrus, the right middle frontal gyrus, and the left triangular part of inferior frontal gyrus in 3D brain were showed. **(B,C)** The changes of metabolic connectivity among different brain regions were showed. The changes in metabolic connectivity of the left precentral gyrus, the left middle frontal gyrus, the right middle frontal gyrus, and the left triangular part of inferior frontal gyrus with other brain regions were annotated in panel **(B)**, and labeled with red font in panel **(C)**. The different number indicate different brain region, which was interpreted in Supplementary Table 3. The color bar indicates the absolute value of magnitude changes in connectivity strength in panel **(B)** and magnitude changes in connectivity strength in panel **(C)**. *N* = 165 cases for control patients. *N* = 56 cases for T2DM group.

To analyze the brain network connectivity, we selected 165 cases of control patients, who had similar average ages (*p* = 0.6517) and sex compositions (*p* = 0.955) with patients with T2DM. Using BrainNet Viewer, we analyzed the brain network metabolic connectivity of patients with T2DM. We found most of the metabolic connections among the 90 brain regions were weakened in patients with T2DM. The metabolic connections between the left middle frontal gyrus, or the left triangular part of inferior frontal gyrus, with other brain regions were also weakened (Figures 6A–C). But the metabolic connections between left precentral gyrus, or the right middle frontal gyrus, with other brain regions were unchanged (Figures 6A–C).

### 3.8 Brain neurometabolism association to clinical cues in obese patients

**Figure 7:**
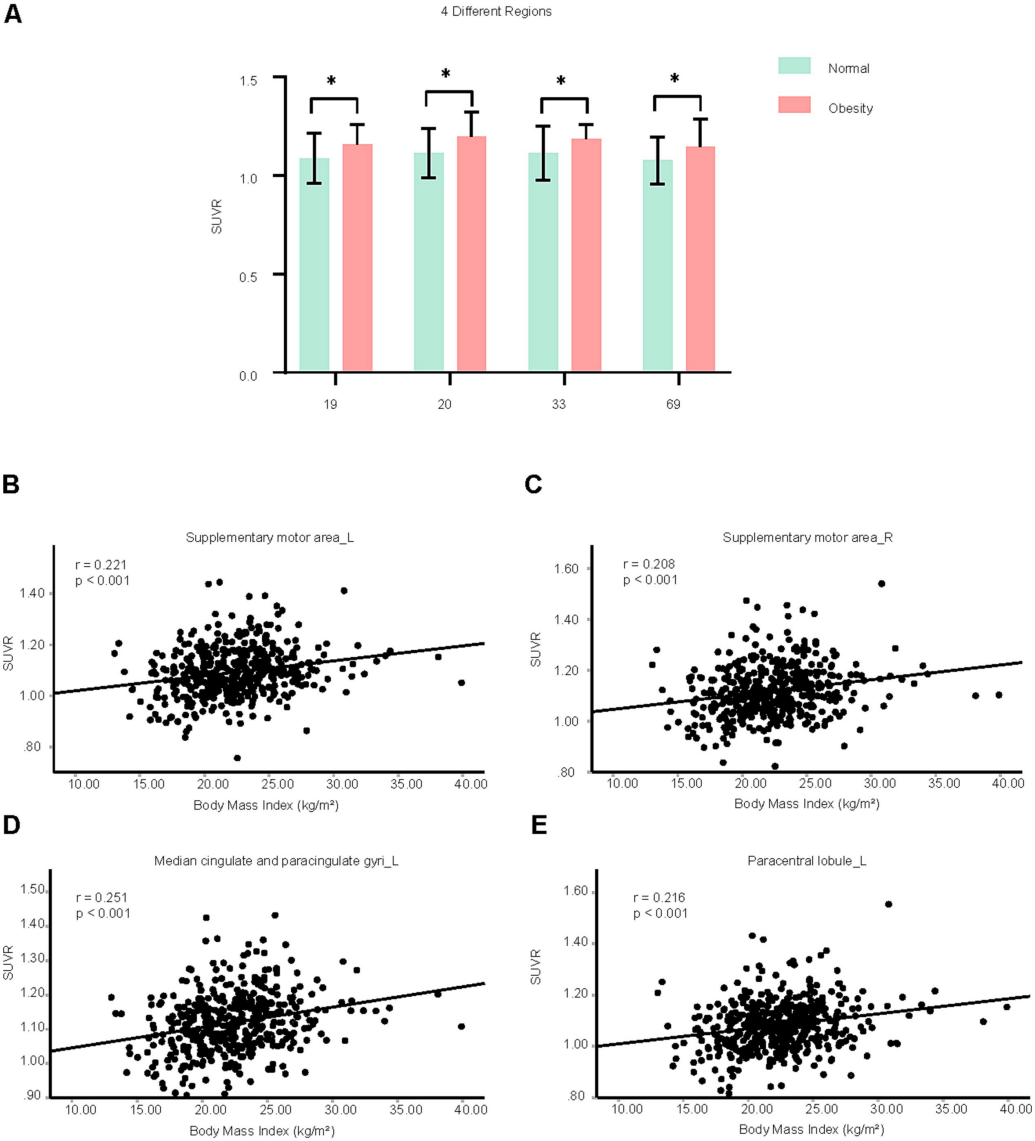
Brain neurometabolism association to clinical cues in obese patients. **(A)** The SUVRs of different brain regions of control and obese patients were compared using two-tailed unpaired Student’s t test or two-tailed independent Student’s t-test with Welch’s correction. The different number indicate different brain region, which was interpreted in Supplementary Table 3. *N* = 497 cases for control patients. *N* = 11 cases for obese group. **(B–E)** The correlations between the SUVRs of different brain regions and BMI in control and obese patients were analyzed using partial correlation analysis by setting age, sex as control variables. *N* = 446 cases. Data shown were mean ± SD. *p < 0.05.

The average age (*p* = 0.2626) and the sex compositions (*p* = 0.652) were similar between obese group and control patients. Compared with control patients, the SUVRs of 4 brain regions in the obese group were significantly increased, including bilateral supplementary motor area, left median cingulate and paracingulate gyri, and left paracentral lobule (Figure 7A). Moreover, the SUVRs of these four brain regions were positively correlated with BMI (Figures 7B–E).

But there were no correlations between the SUVRs of these four brain regions and blood lipid examination, including triglycerides, total cholesterol, high-density lipoprotein cholesterol, and low-density lipoprotein cholesterol (Supplementary Table 2).

For other blood biochemical indicators in obese patients, the SUVR of left supplementary motor area was positively correlated with urea, but negatively with aspartate aminotransferase (Supplementary Figures 5A–D). The SUVR of right supplementary motor area was positively correlated with uric acid. The SUVR of left paracentral lobule was positively correlated with alkaline phosphatase (Supplementary Figures 5A–D).

### 3.9 Alterations in brain network connectivity in obese patients

**Figure 8:**
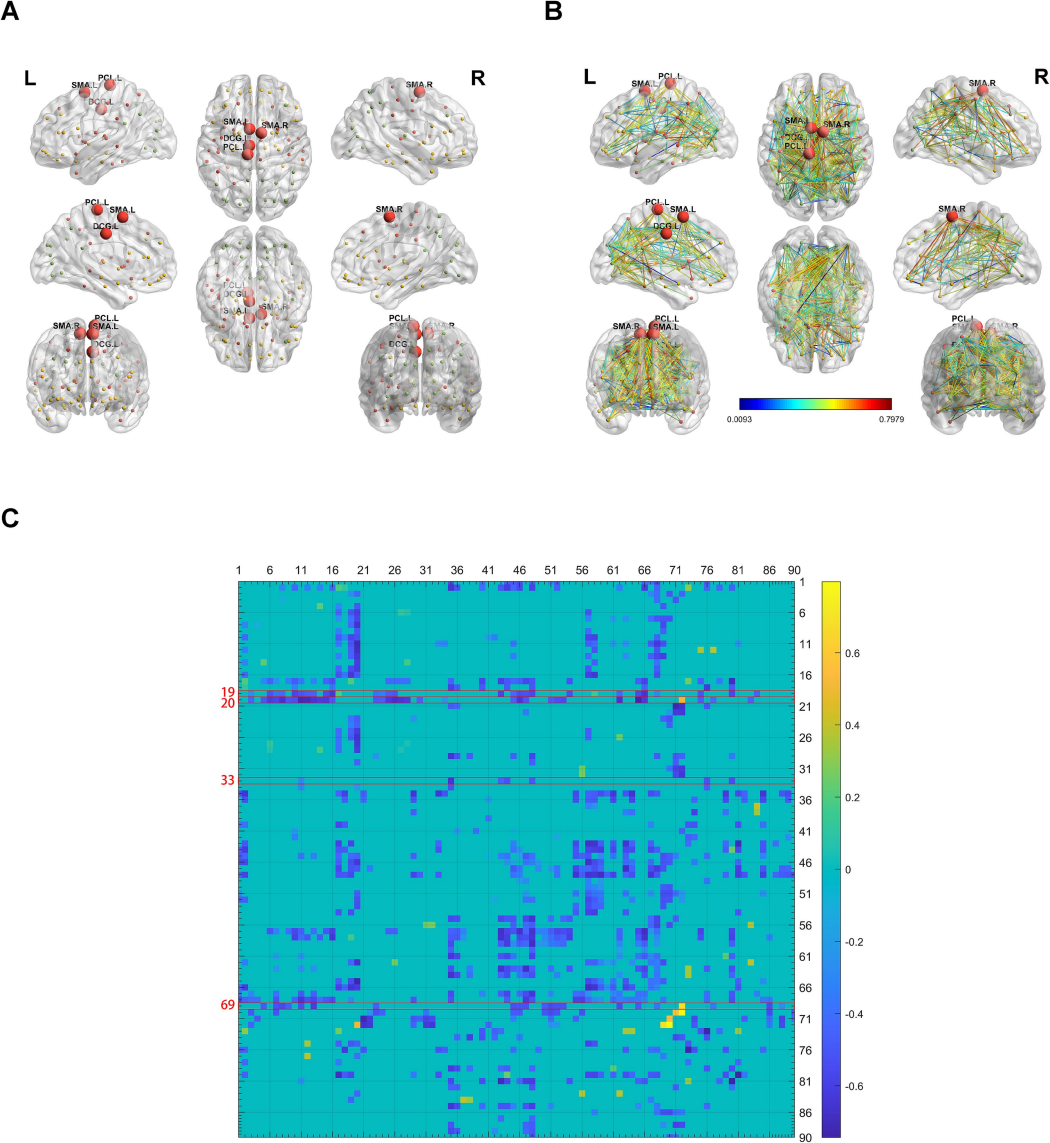
Alterations in brain network connectivity in obese patients. **(A)** Locations of bilateral supplementary motor area, left median cingulate and paracingulate gyri, and left paracentral lobule in 3D brain were showed. **(B,C)** The changes of metabolic connectivity among different brain regions were showed. The changes in metabolic connectivity of bilateral supplementary motor area, left median cingulate and paracingulate gyri, and left paracentral lobule with other brain regions were annotated in panel **(B)**, and labeled with red font in panel **(C)**. The different number indicate different brain region, which was interpreted in Supplementary Table 3. The color bar indicates the absolute value of magnitude changes in connectivity strength in panel **(B)** and magnitude changes in connectivity strength in panel **(C)**. *N* = 497 cases for control patients. *N* = 11 cases for obese group.

Using BrainNet Viewer, we analyzed the brain network metabolic connectivity of obese patients. We found most of the metabolic connections among the 90 brain regions were weakened in obese patients (Figures 8A–C). The metabolic connections between the bilateral supplementary motor area, left median cingulate and paracingulate gyri, or left paracentral lobule, with other brain regions were also weakened (Figures 8A–C).

### 3.10 Brain neurometabolism association to clinical cues in gout patients

Compared with control patients, the average age of gout patients was similar with them (*p* = 0.2072), but there were more males in gout patients (*p* = 0.014). Then we analyzed the corrected SUVRs, which were adjusted according to the age and sex, of patients with gout.

Compared with control patients, the corrected SUVR of right paracentral lobule in the gout group was significantly increased (Supplementary Figure 6A). Among the blood biochemical indicators, the SUVR of right paracentral lobule was negatively correlated with fasting blood-glucose, glutamic oxalacetic transaminase, total bilirubin and alkaline phosphatase (Supplementary Figures 6B–E and Supplementary Table 2).

### 3.11 Alterations in brain network connectivity in gout patients

To analyze the brain network connectivity, we selected 14 cases of control patients, who had similar average ages (*p* = 0.99) and sex compositions (*p* = 1) with patients with gout. Using BrainNet Viewer, we analyzed the brain network metabolic connectivity of gout patients at *S* = 0.55. We found most of the metabolic connections among the 90 brain regions were enhanced in gout patients (Supplementary Figures 7A–C). The metabolic connections between right paracentral lobule with other brain regions were also enhanced (Supplementary Figures 7A–C). The metabolic connections between most brain regions were also enhanced in the gout group compared to the normal group. Then we analyzed the brain network metabolic connectivity of gout patients at *S* = 0.35 (Supplementary Figure 8A and Supplementary Table 5), and we found the network connectivity patterns were consistent across both sparsity levels.

## 4 Discussion

The brain plays a pivotal role in regulating physiological and mental activities to maintain body homeostasis. It governs eating behavior, food selection, digestion, absorption, and energy metabolism, suggesting its critical involvement in the progression of metabolic diseases. Obesity is associated with an increased risk of other metabolic disorders, and comorbidities such as hypertension and T2DM frequently co-occur. Patients with metabolic diseases frequently experience complications during disease progression, indicating potential interconnections among these conditions that may be mediated by the brain.

Our study found the SUVRs of frontal lobe, occipital lobe, temporal lobe, bilateral insula, bilateral cuneus, left pallidum, and left caudate nucleus were decreased, and the SUVR of right amygdala was increased in hypertension group. The SUVRs of the frontal lobe, cingulate, occipital, parietal lobes, cuneus, precuneus, caudate nucleus, and thalamus were decreased in T2DM group. The SUVRs of the bilateral supplementary motor area, left median cingulate and paracingulate gyri, and left paracentral lobule were significantly increased in obese group. The SUVR of right paracentral lobule in the gout group was significantly increased. Most of these brain regions are related with cognitive function, including cuneus, precuneus (Mattioli et al., 2021), cingulate lobes (Sheth et al., 2012), caudate nucleus (Grahn et al., 2008). The decreased SUVRs of these brain regions imply the possible cognitive deficits in patients with hypertension or T2DM. Amygdala is an important region related with chronic stress-induced hypertension (Sheng et al., 2023). The increased SUVR of right amygdala in hypertension group implies the possible association with high blood pressure. The activation of glucose-responsive neurons of the paraventricular thalamus increases motivated sucrose-seeking behavior and these neurons may be associated with sugar overconsumption in obesity and T2DM (Labouèbe et al., 2016). The decreased SUVR of thalamus implies the possible feedback regulation in patients with T2DM. Interestingly, ROI-based volumetric analysis has identified a reduction in thalamic volume in patients with T2DM. Moreover, a decline in cognitive processing speed has been associated with more severe gray matter atrophy in the bilateral dorsomedial thalamus (Chen et al., 2017). The observed reductions in both SUVR and thalamic volume in T2DM patients suggest potential neurodegenerative processes affecting this region.

Our study establishes a link between neurometabolism and peripheral clinical biomarkers. In hypertension group, blood pressure-related brain regions, including left cuneus, left superior occipital gyrus, right superior parietal gyrus, might also be associated with blood uric acid, creatinine, K^+^, and apolipoprotein B. What’s more, the level of blood creatinine was increased significantly in hypertension group. Compared with healthy controls, minimal nephro-encephalopathy shows decreased long-range functional connectivity density in superior parietal gyrus (Zhang et al., 2015). The right superior parietal gyrus might be involved in the regulation of renal function in patients with hypertension. In patients with T2DM, blood glucose-related brain regions, including left precentral gyrus, bilateral middle frontal gyrus and left precentral gyrus, might also be associated with blood triglycerides and cholinesterase. The triglyceride was significantly higher in pre-T2DM and also T2DM, compared to normoglycemia (Jasim et al., 2022). Triglyceride–glucose index is an easy, cheap, and available marker for detection of microvascular complications in patients with T2DM (Kassab et al., 2023). The left precentral gyrus and bilateral middle frontal gyrus might be the important brain regions that associate with the progression of T2DM. In the obese patients, bilateral supplementary motor area and left paracentral lobule might regulate urea nitrogen, aspartate aminotransferase, and alkaline phosphatase, but not blood lipid level. The result implies the possible association of obesity with liver and renal function. In gout patients, right paracentral lobule might be associated with fasting blood-glucose, glutamic oxalacetic transaminase, total bilirubin and alkaline phosphatase. Frequent gout flares have a good predictive capability toward NAFLD development and played a synergistic role in the development of NAFLD (Si et al., 2023). The right paracentral lobule may be associated with NAFLD development in gout patients.

Our study also implies the differences of brain network metabolic connectivity among different metabolic disease. The brain network metabolic connectivity was decreased in patients with hypertension, T2DM, and obesity. But the brain network metabolic connectivity was increased in gout patients. The metabolic brain network connectivity and global efficiency are increased with aging from infant to adolescent (Huang et al., 2020). Aging is associated with weakened functional connectivity as established by functional MRI (Geerligs et al., 2015). The metabolic connectivity was decreased in Alzheimer’s disease and dementia with Lewy bodies (Imai et al., 2020). However, high blood pressure (Lande and Kupferman, 2019), high BMI (Tanaka et al., 2020), and T2DM (Biessels and Despa, 2018) increase the mental problems and decrease the cognitive function in human. On the contrary, gout or hyperuricemia might have a protective effect against Alzheimer’s disease (Wang et al., 2023). It might be possible that hyperuricemia is associated with improved brain network metabolic connectivity although the samples are small. The conclusion would be more credible if more gout cases were involved.

There were few patients without malignant tumors performing PET-CT examination. And many other examinations are also incomplete in some patients. Thus, the sample size was limited. And it’s difficult to further subgroup patients with hypertension, or T2DM, or obesity, or gout, according to the different duration, classification, and treatment of diseases. Otherwise, more interesting analysis would be done to suppose the possible role of brain on the progression of metabolic diseases. Despite these findings, our study has several notable limitations. First, the control group included individuals with a history of pneumonia, thyroid cancer, hepatitis B, or limb fractures. Although we implemented stringent inclusion and exclusion criteria to minimize confounding effects, it remains possible that conditions such as pneumonia, hepatitis B, and thyroid cancer history may have influenced brain metabolism to some extent. Second, our study was exploratory in nature, and we employed two-tailed independent Student’s t-tests without corrections for multiple comparisons to analyze SUVR differences between groups. While this approach allowed us to identify potentially significant and meaningful brain regions, it also increased the risk of Type I errors. Third, all patients in the T2DM group were receiving regular metformin treatment. Given that metformin may influence brain metabolism, this group should be considered as a treated-T2DM cohort rather than a treatment-naïve population. Fourth, our study primarily conducted correlational analyses, which do not establish causal relationships between metabolic alterations and neural changes. Fifth, over 80% of patients in our study lacked concurrent MRI scans, limiting our ability to integrate PET and MRI findings for a more comprehensive investigation. Interestingly, a study by Eithan Kotkowski examined gray matter volume (GMV) alterations associated with metabolic syndrome (MetS) components using MRI-based volumetric analysis (Kotkowski et al., 2022). Their findings revealed structural atrophy across multiple brain regions and they identified waist circumference emerging as the strongest predictor of GMV reductions. In contrast, our study employed PET-CT imaging to assess brain metabolic activity, providing a functional perspective on how metabolic disorders relate with neural metabolism. Our PET-CT approach offers insights into functional metabolic alterations, complementing the structural findings of the neural signature of MetS (NS-MetS) study. Specifically, our results suggest that the right superior parietal gyrus may be implicated in renal dysfunction during hypertension progression, while the left precentral gyrus and bilateral middle frontal gyri may be associated with dyslipidemia and atherosclerotic cardiovascular disease risk in T2DM. Furthermore, our findings indicate that gout exhibits distinct metabolic patterns compared to other metabolic diseases, potentially suggesting a neuroprotective effect-a perspective not explored within the NS-MetS framework. Future research integrating both structural and functional imaging modalities may provide a more refined understanding of the interplay between metabolic dysregulation and brain function. Lastly, there are only a small sample size of gout patients in our study and the findings from gout patients should be interpreted with caution and considered exploratory. Future studies with larger, more balanced cohorts are needed to validate these results.

In summary, uric acid may be negatively associated with blood pressure and glucose levels, while blood glucose and lipid levels may be positively correlated. Gout appears distinct from other metabolic diseases and may offer a protective effect on brain function. The right superior parietal gyrus might be associated with both blood pressure and renal function. The left precentral gyrus and bilateral middle frontal gyrus might be associated with both fasting blood-glucose and triglycerides, implying the possible association of these brain regions with the dyslipidemia and potential atherosclerotic cardiovascular disease in patients with T2DM. Our study indicates potential connections among these metabolic diseases and the involvement of the brain in their progression.

## Data Availability

The raw data supporting the conclusions of this article will be made available by the authors without undue reservation.

## Footnotes

^1^ https://github.com/IHEP-Brain-Imaging/Spatial-Normalization-of-Brain-PET-Images

^2^https://www.nitrc.org/projects/wfu_pickatlas, version 3.0.5.

## Supplementary material

SUPPLEMENTARY FIGURE 1 The neurometabolism of multiple brain regions were decreased in patients with hypertension. **(A)** The SUVRs of different brain regions of control and hypertension patients were compared using two-tailed unpaired Student’s t test or two-tailed independent Student’s t-test with Welch’s correction. The different number indicate different brain region, which was interpreted in Supplementary Table 3. *N* = 497 cases for control patients. *N* = 112 cases for hypertension group. Data shown were mean ± SD. **p* < 0.05; **, *p* < 0.01; ***, *p* < 0.001.

SUPPLEMENTARY FIGURE 2 The SUVR of left cuneus was positively correlated with uric acid in control patients. **(A–E)** The value of serum potassium (*N* = 332 cases for control patients; *N* = 68 cases for hypertension group), creatinine (*N* = 348 cases for control patients; *N* = 71 cases for hypertension group), uric acid (*N* = 119 cases for control patients; *N* = 22 cases for hypertension group), urea nitrogen/creatinine (*N* = 314 cases for control patients; *N* = 56 cases for hypertension group), and apolipoprotein B (*N* = 162 cases for control patients; *N* = 26 cases for hypertension group) were compared using two-tailed unpaired Student’s t test or two-tailed independent Student’s t-test with Welch's correction between control and hypertension group. **(F,G)** The correlations between SUVR of left cuneus and uric acid (**F**, *N* = 99 cases) or urea nitrogen/creatinine (**G**, *N* = 275 cases) in control patients were analyzed using partial correlation analysis by setting age, sex and BMI as control variables. **(H–J)** The correlations between SUVR of left superior occipital gyrus and serum potassium (**H**, *N* = 290 cases), urea nitrogen/creatinine (**I**, *N* = 275 cases) or apolipoprotein B (**J**, *N* = 145 cases) in control patients were analyzed using partial correlation analysis by setting age, sex and BMI as control variables. **(K–L)** The correlations between SUVR of right superior parietal gyrus and creatinine (**K**, *N* = 302 cases), or uric acid (**L**, *N* = 99 cases) in control patients were analyzed using partial correlation analysis by setting age, sex and BMI as control variables. Data shown were mean ± SD. **p* < 0.05.

SUPPLEMENTARY FIGURE 3 The neurometabolism of multiple brain regions were decreased in patients with T2DM. **(A)** The SUVRs of different brain regions of control and T2DM patients were compared using two-tailed unpaired Student’s t test or two-tailed independent Student’s t-test with Welch’s correction. The different number indicate different brain region, which was interpreted in Supplementary Table 3. *N* = 497 cases for control patients. *N* = 56 cases for T2DM group. Data shown were mean ± SD. **p* < 0.05; ***p* < 0.01; ****p* < 0.001.

SUPPLEMENTARY FIGURE 4 The SUVR of left precentral gyrus was negatively correlated with fasting blood-glucose in control patients. **(A–D)** The correlations between SUVRs of left precentral gyrus, left middle frontal gyrus, right middle frontal gyrus or left Inferior frontal gyrus and fasting blood-glucose in control patients were analyzed using partial correlation analysis by setting age, sex and BMI as control variables. *N* = 292 cases.

SUPPLEMENTARY FIGURE 5 The correlations between SUVRs of different brain regions and blood biochemical indicators in obese patients. **(A–D)** The correlations between the SUVRs of bilateral supplementary motor area, left median cingulate and paracingulate gyri, or left paracentral lobule and blood biochemical indicators in obese patients were analyzed using partial correlation analysis by setting age, sex and BMI as control variables. *N* = 8 cases in panels **(A,B)**, 6 cases in panels **(C,D)**.

SUPPLEMENTARY FIGURE 6 Brain neurometabolism association to clinical cues in gout patients. **(A)** The corrected SUVR of right paracentral lobule of control and gout patients was compared using two-tailed unpaired Student’s t test. *N* = 497 cases for control patients. *N* = 14 cases for gout group. **(B–E)** The correlations between SUVR of right paracentral lobule and blood biochemical indicators in control and gout patients were analyzed using partial correlation analysis by setting age, sex and BMI as control variables. *N* = 299 cases in panel **(B)**, 259 cases in panel **(C)**, 234 cases in panel **(D)**, 245 cases in panel **(E)**. Data shown were mean ± SD. ***p* < 0.01.

SUPPLEMENTARY FIGURE 7 Alterations in brain network connectivity in gout patients. **(A)** Location of right paracentral lobule in 3D brain was showed. **(B,C)** The changes of metabolic connectivity among different brain regions were showed when *S* = 0.55. The changes in metabolic connectivity of right paracentral lobule with other brain regions were annotated in panel **(B)**, and labeled with red font in panel **(C)**. The different number indicated different brain region, which was interpreted in Supplementary Table 3. The color bar indicates the absolute value of magnitude changes in connectivity strength in panel **(B)** and magnitude changes in connectivity strength in panel **(C)**. *N* = 14 cases for control patients. *N* = 14 cases for gout group.

SUPPLEMENTARY FIGURE 8 Alterations in brain network connectivity in gout patients. **(A)** The changes of metabolic connectivity among different brain regions were showed when *S* = 0.35. The different number indicated different brain region, which was interpreted in Supplementary Table 3. The color bar indicates magnitude changes in connectivity strength. *N* = 14 cases for control patients. *N* = 14 cases for gout group.
