# Supplementary figures and images for: Relations between neurometabolism and clinical biomarkers in patients with metabolic disease
Source: Front Neurosci. 2025 Apr 15;19:1547010. doi: 10.3389/fnins.2025.1547010 (PMC12037510; doi:10.3389/fnins.2025.1547010)

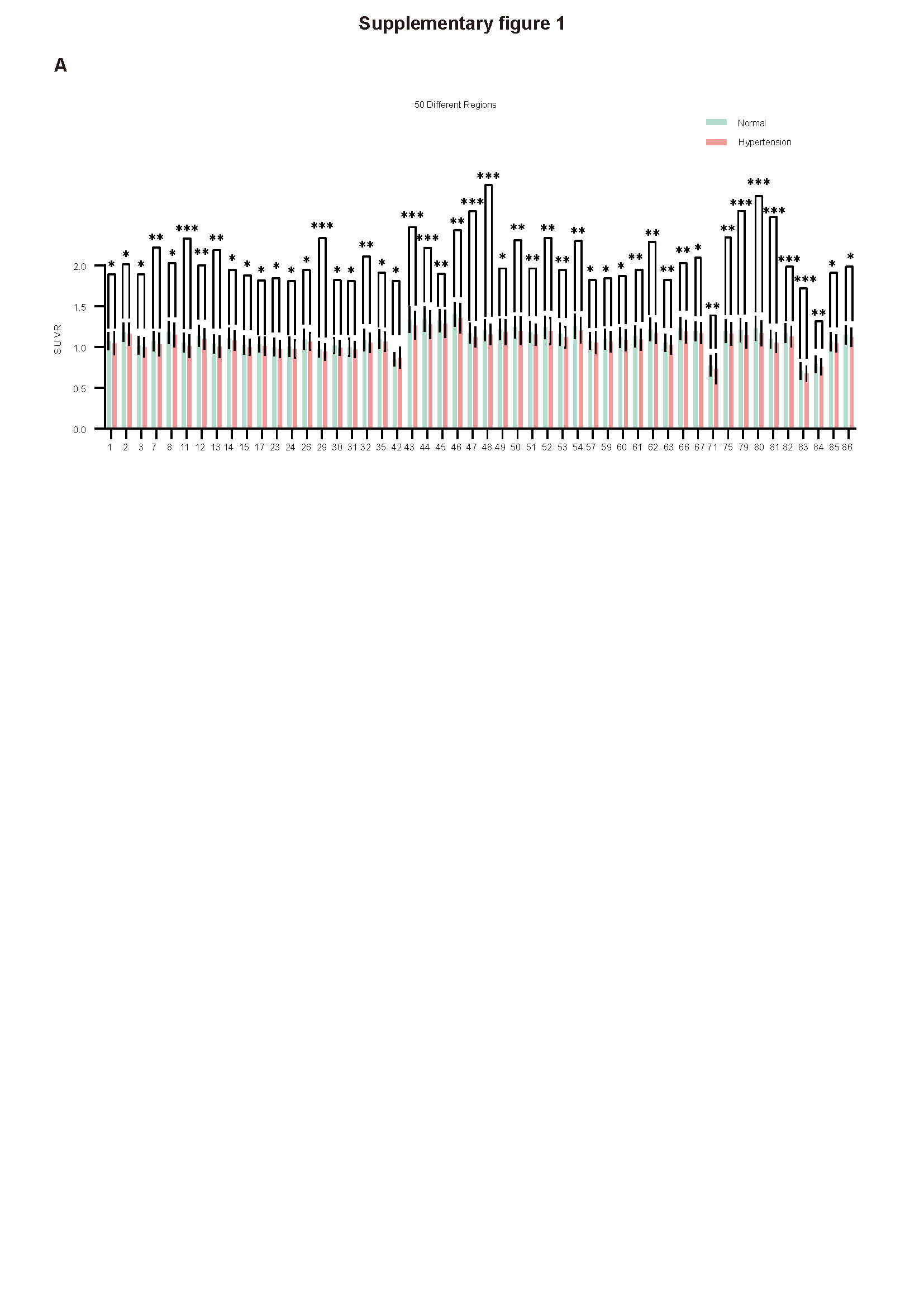

Supplement: SUPPLEMENTARY FIGURE 1 — The neurometabolism of multiple brain regions were decreased in patients with hypertension. (A) The SUVRs of different brain regions of control and hypertension patients were compared using two-tailed unpaired Student’s t test or two-tailed independent Student’s t-test with Welch’s correction. The different number indicate different brain region, which was interpreted in Supplementary Table 3. N = 497 cases for control patients. N = 112 cases for hypertension group. Data shown were mean ± SD. *p < 0.05; **, p < 0.01; ***, p < 0.001. [file Image_1.TIFF]

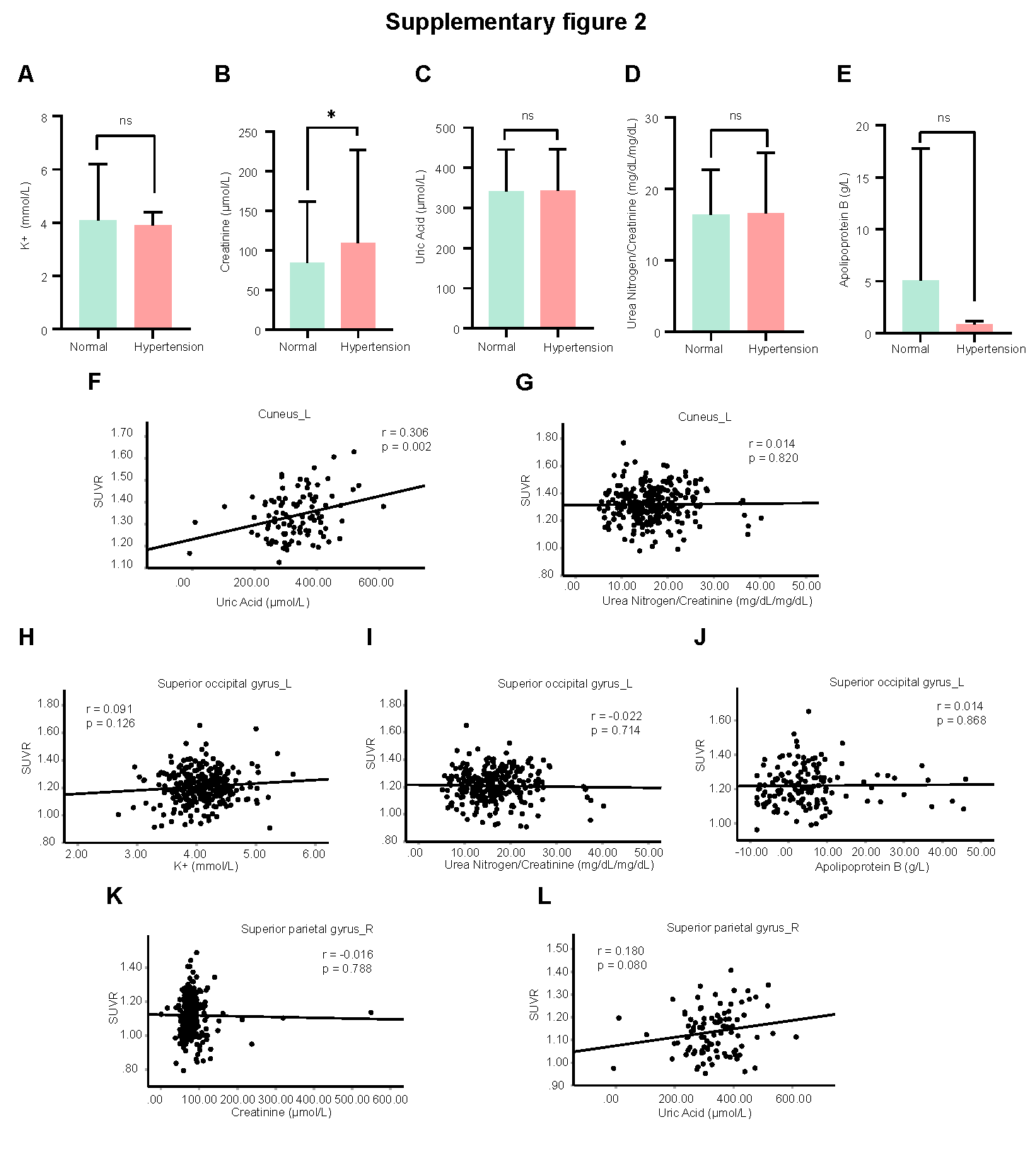

Supplement: SUPPLEMENTARY FIGURE 2 — The SUVR of left cuneus was positively correlated with uric acid in control patients. (A–E) The value of serum potassium (N = 332 cases for control patients; N = 68 cases for hypertension group), creatinine (N = 348 cases for control patients; N = 71 cases for hypertension group), uric acid (N = 119 cases for control patients; N = 22 cases for hypertension group), urea nitrogen/creatinine (N = 314 cases for control patients; N = 56 cases for hypertension group), and apolipoprotein B (N = 162 cases for control patients; N = 26 cases for hypertension group) were compared using two-tailed unpaired Student’s t test or two-tailed independent Student’s t-test with Welch's correction between control and hypertension group. (F,G) The correlations between SUVR of left cuneus and uric acid (F, N = 99 cases) or urea nitrogen/creatinine (G, N = 275 cases) in control patients were analyzed using partial correlation analysis by setting age, sex and BMI as control variables. (H–J) The correlations between SUVR of left superior occipital gyrus and serum potassium (H, N = 290 cases), urea nitrogen/creatinine (I, N = 275 cases) or apolipoprotein B (J, N = 145 cases) in control patients were analyzed using partial correlation analysis by setting age, sex and BMI as control variables. (K–L) The correlations between SUVR of right superior parietal gyrus and creatinine (K, N = 302 cases), or uric acid (L, N = 99 cases) in control patients were analyzed using partial correlation analysis by setting age, sex and BMI as control variables. Data shown were mean ± SD. *p < 0.05. [file Image_2.TIFF]

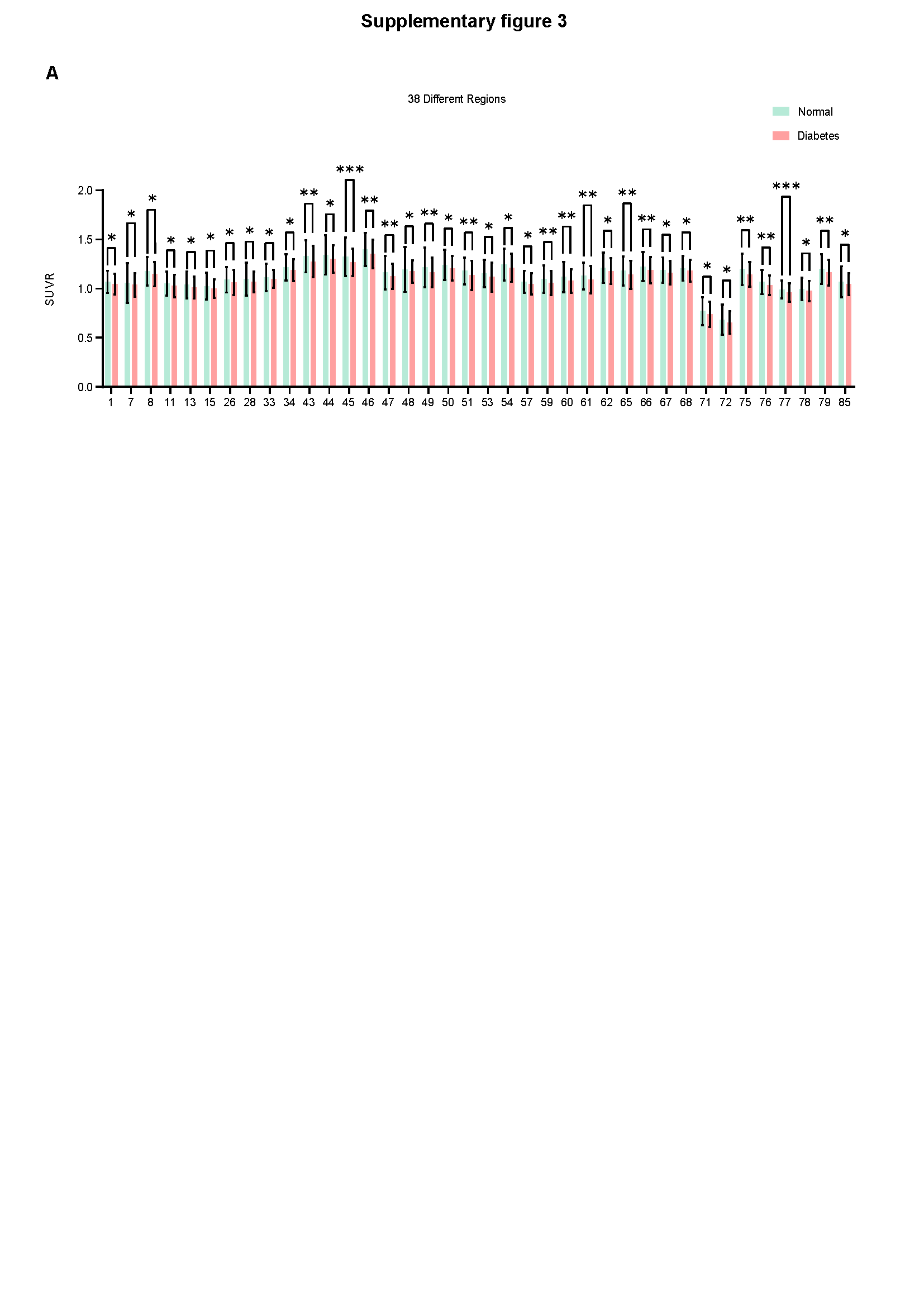

Supplement: SUPPLEMENTARY FIGURE 3 — The neurometabolism of multiple brain regions were decreased in patients with T2DM. (A) The SUVRs of different brain regions of control and T2DM patients were compared using two-tailed unpaired Student’s t test or two-tailed independent Student’s t-test with Welch’s correction. The different number indicate different brain region, which was interpreted in Supplementary Table 3. N = 497 cases for control patients. N = 56 cases for T2DM group. Data shown were mean ± SD. *p < 0.05; **p < 0.01; ***p < 0.001. [file Image_3.TIFF]

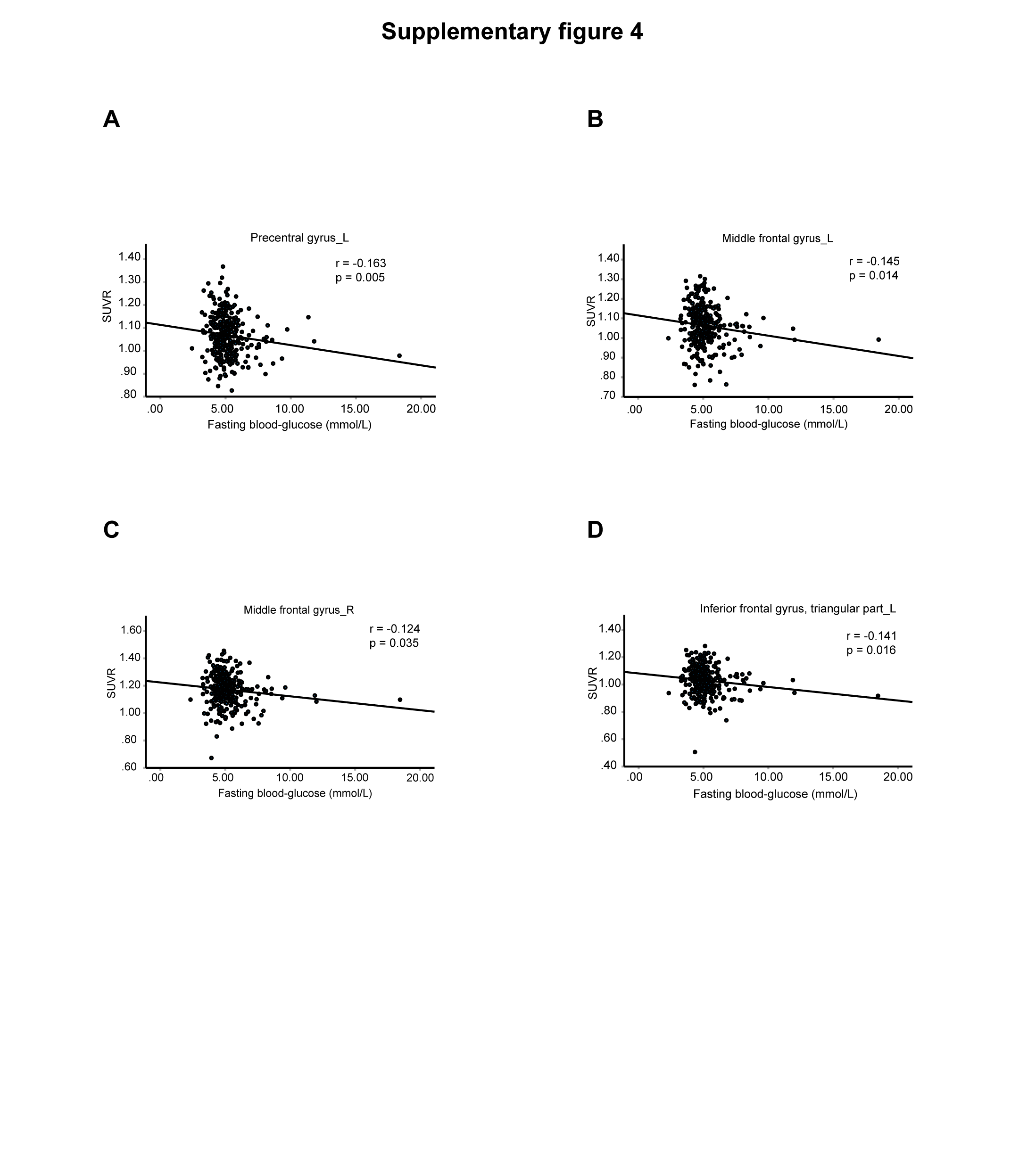

Supplement: SUPPLEMENTARY FIGURE 4 — The SUVR of left precentral gyrus was negatively correlated with fasting blood-glucose in control patients. (A–D) The correlations between SUVRs of left precentral gyrus, left middle frontal gyrus, right middle frontal gyrus or left Inferior frontal gyrus and fasting blood-glucose in control patients were analyzed using partial correlation analysis by setting age, sex and BMI as control variables. N = 292 cases. [file Image_4.TIF]

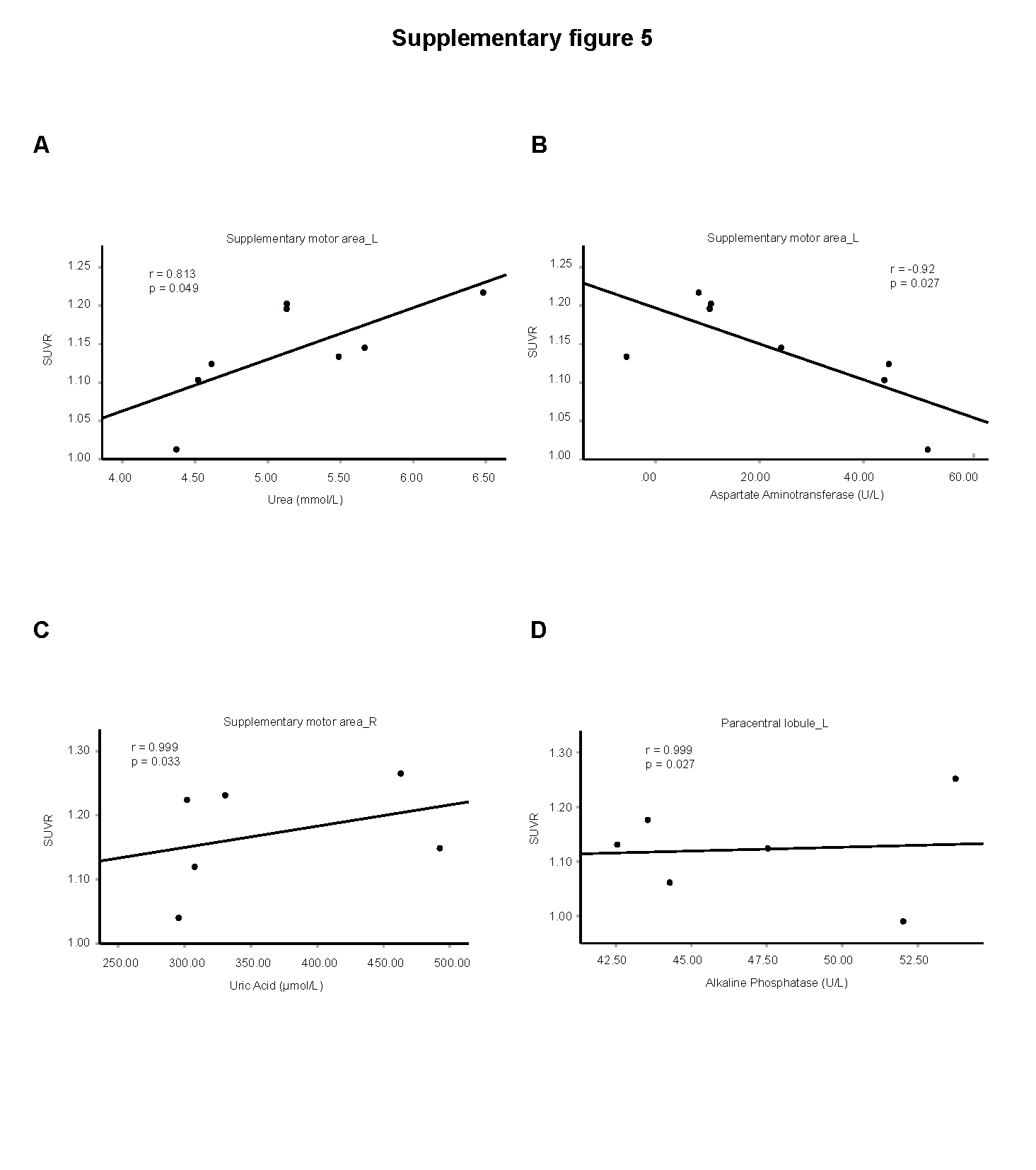

Supplement: SUPPLEMENTARY FIGURE 5 — The correlations between SUVRs of different brain regions and blood biochemical indicators in obese patients. (A–D) The correlations between the SUVRs of bilateral supplementary motor area, left median cingulate and paracingulate gyri, or left paracentral lobule and blood biochemical indicators in obese patients were analyzed using partial correlation analysis by setting age, sex and BMI as control variables. N = 8 cases in panels (A,B), 6 cases in panels (C,D). [file Image_5.TIFF]

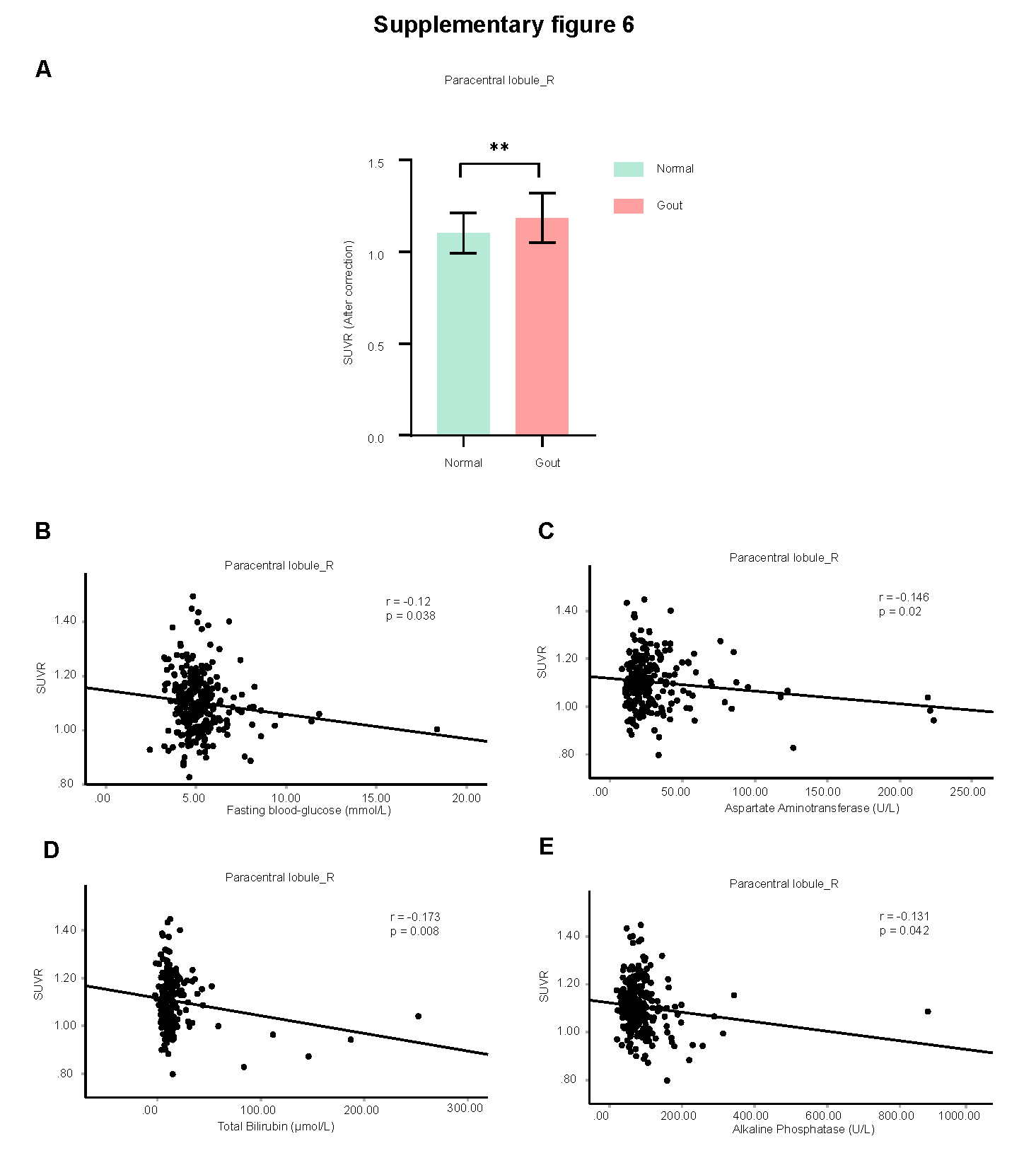

Supplement: SUPPLEMENTARY FIGURE 6 — Brain neurometabolism association to clinical cues in gout patients. (A) The corrected SUVR of right paracentral lobule of control and gout patients was compared using two-tailed unpaired Student’s t test. N = 497 cases for control patients. N = 14 cases for gout group. (B–E) The correlations between SUVR of right paracentral lobule and blood biochemical indicators in control and gout patients were analyzed using partial correlation analysis by setting age, sex and BMI as control variables. N = 299 cases in panel (B), 259 cases in panel (C), 234 cases in panel (D), 245 cases in panel (E). Data shown were mean ± SD. **p < 0.01. [file Image_6.TIFF]

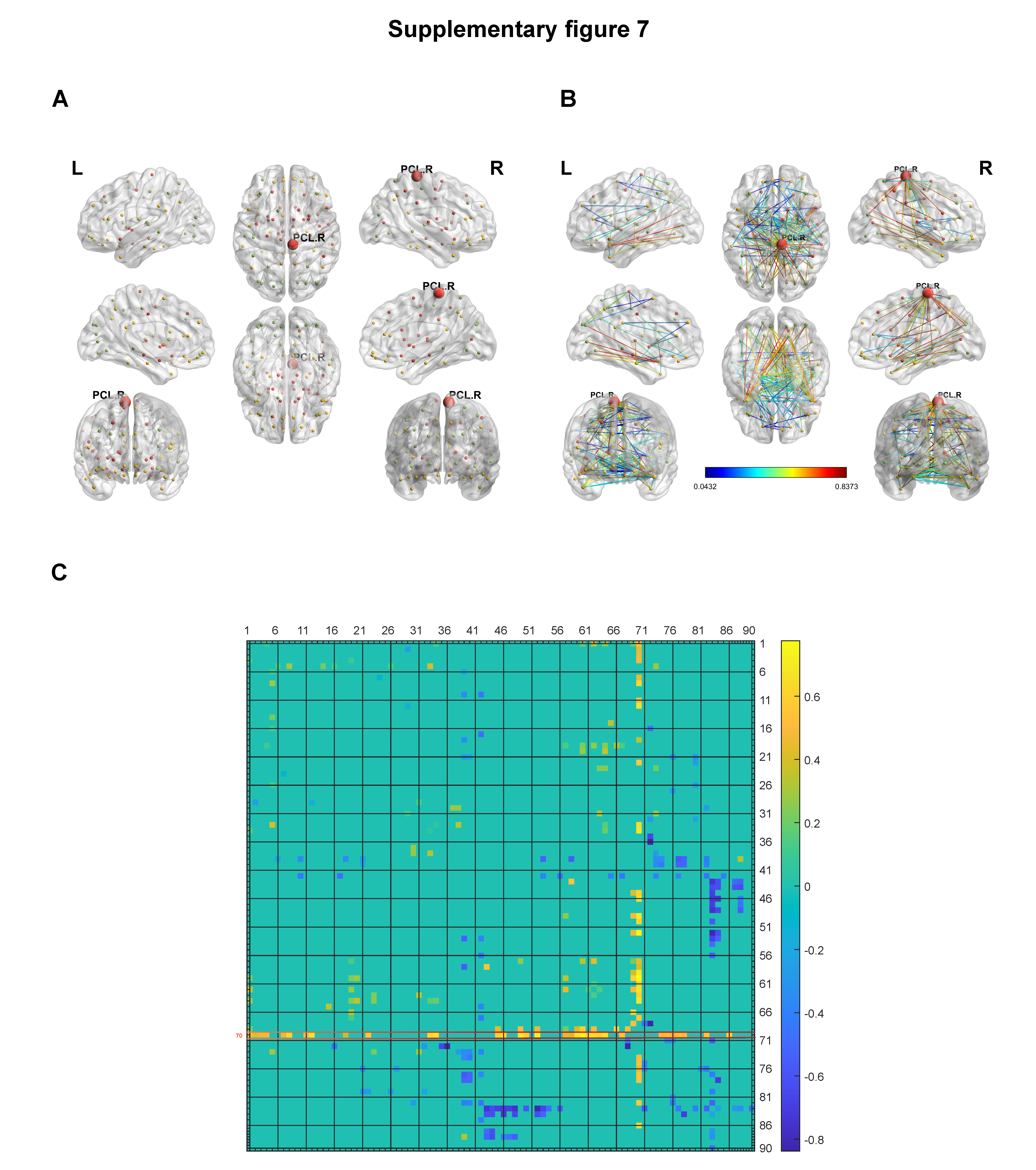

Supplement: SUPPLEMENTARY FIGURE 7 — Alterations in brain network connectivity in gout patients. (A) Location of right paracentral lobule in 3D brain was showed. (B,C) The changes of metabolic connectivity among different brain regions were showed when S = 0.55. The changes in metabolic connectivity of right paracentral lobule with other brain regions were annotated in panel (B), and labeled with red font in panel (C). The different number indicated different brain region, which was interpreted in Supplementary Table 3. The color bar indicates the absolute value of magnitude changes in connectivity strength in panel (B) and magnitude changes in connectivity strength in panel (C). N = 14 cases for control patients. N = 14 cases for gout group. [file Image_7.TIFF]

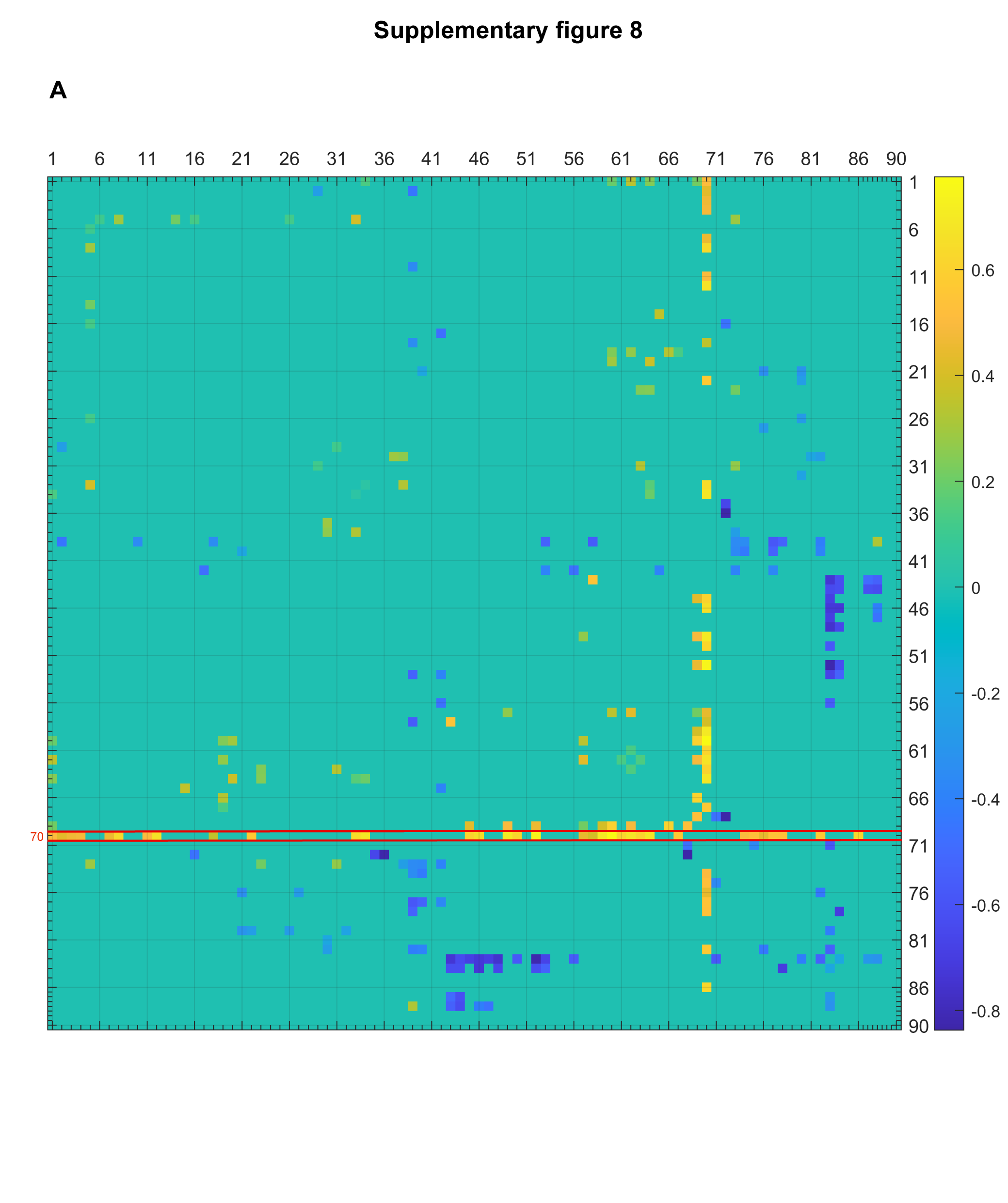

Supplement: SUPPLEMENTARY FIGURE 8 — Alterations in brain network connectivity in gout patients. (A) The changes of metabolic connectivity among different brain regions were showed when S = 0.35. The different number indicated different brain region, which was interpreted in Supplementary Table 3. The color bar indicates magnitude changes in connectivity strength. N = 14 cases for control patients. N = 14 cases for gout group. [file Image_8.TIF]
